# Exploring the Correlation Between Multiple Latent Variables and Covariates in Hierarchical Data Based on the Multilevel Multidimensional IRT Model

**DOI:** 10.3389/fpsyg.2019.02387

**Published:** 2019-10-25

**Authors:** Jiwei Zhang, Jing Lu, Feng Chen, Jian Tao

**Affiliations:** ^1^School of Mathematics and Statistics, Yunnan University, Kunming, China; ^2^School of Mathematics and Statistics, Northeast Normal University, Changchun, China; ^3^Department of East Asian Studies, The University of Arizona, Tucson, AZ, United States

**Keywords:** education assessment, teacher satisfactions, multidimensional item response theory, multilevel model, Bayesian estimation

## Abstract

In many large-scale tests, it is very common that students are nested within classes or schools and that the test designers try to measure their multidimensional latent traits (e.g., logical reasoning ability and computational ability in the mathematics test). It is particularly important to explore the influences of covariates on multiple abilities for development and improvement of educational quality monitoring mechanism. In this study, motivated by a real dataset of a large-scale English achievement test, we will address how to construct an appropriate multilevel structural models to fit the data in many of multilevel models, and what are the effects of gender and socioeconomic-status differences on English multidimensional abilities at the individual level, and how does the teachers' satisfaction and school climate affect students' English abilities at the school level. A full Gibbs sampling algorithm within the Markov chain Monte Carlo (MCMC) framework is used for model estimation. Moreover, a unique form of the deviance information criterion (DIC) is used as a model comparison index. In order to verify the accuracy of the algorithm estimation, two simulations are considered in this paper. Simulation studies show that the Gibbs sampling algorithm works well in estimating all model parameters across a broad spectrum of scenarios, which can be used to guide the real data analysis. A brief discussion and suggestions for further research are shown in the concluding remarks.

## 1. Introduction

With the increasing interest in multidimensional latent traits and the advancement in estimation techniques, multidimensional item response theory (IRT) has been developed vigorously which made the model estimation become easy to implement and effective. Single-level multidimensional IRT (MIRT) models were proposed decades ago, as it have the primary features of modeling the correlations among multiple latent traits and categorical response variables (Mulaik, 1972; Reckase, 1972, 2009; Sympson, 1978; Whitely, 1980a,b; Way et al., 1988; Ackerman, 1989; Muraki and Carlson, 1993; Kelderman and Rijkes, 1994; Embretson and Reise, 2000; Béguin and Glas, 2001; Yao and Schwarz, 2006). The MIRT models later incorporated covariates to elucidate the connection between multiple latent traits and predictors (Adams et al., 1997; van der Linden, 2008; De Jong and Steenkamp, 2010; Klein Entink, 2009; Klein Entink et al., 2009; Höhler et al., 2010; Lu, 2012; Muthén and Asparouhov, 2013).

It has become frequent practice to regard IRT model calibration's latent ability as a dependent variable in resulting regression analysis in relation to educational and psychological measurement. Measurement error within latent ability estimates is ignored in this two-stage treatment resulting in statistical inferences that may be biased. Specially, measurement error can reduce the statistical power of impact studies and deteriorate the researchers' ability to ascertain relationships among different variables affecting student outcomes (Lu et al., 2005). One error that can reduce the statistical capabilities of impact studies and make it difficult for researchers to identify relationships between variables related to student outcomes is the measurement error.

Taking a multilevel perspective on item response modeling can avoid issues that arise when analysts use latent regression (using latent variables as outcomes in regression analysis) (Adams et al., 1997). The student population distribution is commonly handled as a between-student model with the IRT model being placed at the lowest level as a within-subject model within the structure of multilevel or hierarchical models. Using a multilevel IRT model gives analysts the ability to estimate item and ability parameters along with structural multilevel model parameters at the same time (e.g., Adams et al., 1997; Kamata, 2001; Hox, 2002; Goldstein, 2003; Pastor, 2003). This results in measurement error associated with estimated abilities being accounted for when estimating the multilevel parameters (Adams et al., 1997).

Although the multilevel IRT models have been deeply studied in the last 20 years, there are significant differences between our multilevel IRT models and the existing literatures in the problem to be solved and the viewpoint of modeling. Next, we discuss the differences from many aspects. Multidimensional IRT models that have a hierarchical structure relationship between specific ability and general ability were developed in 2007 by Sheng and Wikle. Specifically, general ability has a linear relationship with specific ability, or all specific abilities linearly combine within a general ability. However, the hierarchical structure in our study refers to the nested data structure, for example, the students are nested in classes while classes are nested in schools, rather than the hierarchical relationships between specific ability and general ability. The modeling method similar to Sheng and Wikle (2007) also includes Huang and Wang (2014) and Huang et al. (2013). Note that in Huang and Wang (2014), not only the hierarchical abilities models are discussed, but also the multilevel data are modeled. Muthén and Asparouhov (2013) proposed the multilevel multidimensional IRT models to investigate elementary student aggressive-disruptive behavior in school classrooms and the model parameters were estimated in Mplus (Muthén and Muthén, 1998) using Bayes. Although Muthén and Asparouhov (2013) and our current study also focus on the multilevel multidimensional IRT modeling, there are great differences in the model construction. In the multilevel modeling, they suggested that the ability (factor) of each dimension has between-and within-cluster variations. However, the sources of the between—and within—cluster variations are not taken into account. More specifically, whether these two types of variation are affected by the between cluster covariates and within individual background variables have not been further analyzed. Similarly, in the works of both Höhler et al. (2010) and Lu (2012) demonstrated the same modeling method. In our study, the between—and within—cluster variations are further explained by considering the effects of individual and school covariates on multiple dimensional latent abilities. For example, we can consider whether the gender difference between male and female has an important influence on the vocabulary cognitive ability and reading comprehension ability. Moreover, Chalmers (2015) proposed an extended mixed-effects IRT models to analyze PISA data. By using a Metropolis-Hastings Robbins-Monro (MH-RM) stochastic imputation algorithm (cf. Cai, 2010a,b,c, 2013), it evaluates fixed and random coefficients. Rather than directly explaining the multiple dimensional abilities, the individual background (level-1) and school (level-2) covariates are used to model the fixed effects.

In order to illustrate the interactions between unidimensional ability and individual—and school—level covariates where the ability parameters possess a hierarchical nesting structure, Fox and Glas (2001) and Kamata (2001) proposed multilevel IRT models. In this current research, we broaden Fox and Glas (2001) and Kamata (2001)'s models by swapping their unidimensional IRT model with a multidimensional normal ogive model because we want to assess students' four types of abilities from a large-scale English achievement test. We particularly pay attention to investigating the connection between multiple latent traits and covariates. Taking the proposed multilevel multidimensional IRT models as the basis, the following issues will be addressed. (1) According to the model selection results, which model is the best to fit the data and how can judge the individual-level regression coefficients be judged as fixed effect or random effect? (2) How will students from different ends of the socioeconomic-status (SES) score in English performance as tested in four types of latent abilities, based on the level-2 gender (GD), level-3 teacher satisfaction (ST) and school climate (CT) [The details of the Likert questionnaires for measuring teacher satisfaction and school climate, please refer to (Shalabi, 2002)]. (3) What relationship exists between males and females' performances in different latent abilities by controlling for SES, ST and CT. (4) What effects, if any, are seen with different teachers' or schools' effects (covariates)? (5) Is it possible to use a measurement tool to determine whether items' factor patterns correlate to the subscales of the test battery? In particular, will the four subtests of the test battery be discernable according to the discrimination parameters on the four dimensions?

The rest of the article is organized as follows. Section 2 presents the detailed development of the proposed multilevel multidimensional IRT models and procedure for hierarchical data. Section 3 provides a Bayesian estimation method to meet computational challenges for the proposed models. Meanwhile, Bayesian model assessment criteria is discussed in section 3. In section 4, simulation studies are conducted to examine the performances of parameter recovery using the Gibbs sampling algorithm. In addition, a real data analysis of the education quality assessment is given in section 5. We conclude this article with a brief discussions and suggestions for further research in section 6.

## 2. Multilevel Multidimensional IRT Model

The model contains three levels. At the first level, a multidimensional normal ogive IRT model is defined to model the relationship between items, persons, and responses. At the second level, personal parameters are predicted by personal-level covariates, such as an individual's social economic status (SES). At the third level, persons are nested within schools, and school-level covariates are included such as school climate and teacher satisfaction.

• The measurement model at level 1 (multidimensional two parameter normal ogive model; Samejima, 1974; McDonald, 1999; Bock and Schilling, 2003)

(2.1) $$\begin{array}{l} p_{ijk}=P\left(Y_{ijk}=1|\theta_{ij},\xi_{k}\right)=\frac{1}{\sqrt{2\pi }}\overset{\eta_{ijk}}{\underset{-\infty }{\int }}e^{-\frac{t^{2}}{2}}dt. \end{array}$$

In terms of notation, let *j* = 1, …, *J* indicate *J* schools (or groups), and within school *j*, there are *i* = 1, …, *n*_*j*_ individuals. The total number of individuals is *n* = *n*_1_ + *n*_2_ +… + *n*_*J*_. *k* = 1, …, *K* indicate the items. In Equation (2.1), *Y*_*ijk*_ denotes the response of the *i*th individual in the *j*th group answering the *k*th item. The corresponding correct response probability can be expressed as *p*_*ijk*_, and ***θ***_*ij*_ denotes a *Q*-dimensional vectors of ability parameters for the *i*th individual in the *j*th group, i.e., $\theta_{ij}={\left(\theta_{ij1},\theta_{ij2},\ldots ,\theta_{ijQ}\right)}^{\prime },$ and $\xi_{k}={\left(a_{k1},a_{k2},\ldots ,a_{kQ},b_{k}\right)}^{\prime }$ denotes the vector of item parameters, in which ***a***_*k*_ = ${\left(a_{k1},a_{k2},\ldots ,a_{kQ}\right)}^{\prime }$ is a vector of discrimination or slope parameters, and *b*_*k*_ is the difficulty or intercept parameter. Let $\eta_{ijk}=\overset{Q}{\underset{q=1}{\sum }}a_{kq}\theta_{ijq}-b_{k}.$ The latent abilities of different dimensions can be explained by individual-level background covariates. Note that the multidimensional IRT model used in this paper actually belongs to the within-items multidimensional IRT model. That is, each item measures multiple dimensional abilities, and each test item has loadings on all these abilities. Unlike the between-items multidimensional IRT model, each item has a unity loading on one dimensional ability and zero loadings on other dimensional abilities. For a further explanation of the model used in this paper, please see Table 1 in the following simulation study 1.

**Table 1:** Estimation of simulated item parameter estimation using Gibbs sampling algorithm in simulation study 1.

	***a***_***k*1**_			***a***_***k*2**_			***b***_***k***_		
**Item**	**True**	**EAP**	**HPDI**	**True**	**EAP**	**HPDI**	**True**	**EAP**	**HPDI**
1	1^*^	1^*^	−	0^*^	0^*^	−	0^*^	0^*^	−
2	0^*^	0^*^	−	1^*^	1^*^	−	0^*^	0^*^	−
3	0.914	0.877	[0.711, 1.044]	0.686	0.672	[0.551, 0.795]	−1.182	−1.154	[−1.327, −1.005]
4	1.102	1.127	[0.915, 1.355]	1.468	1.485	[1.250, 1.717]	0.441	0.426	[0.203, 0.629]
5	2.055	2.046	[1.674, 2.466]	1.428	1.453	[1.214, 1.678]	−1.197	−1.367	[−1.683, −1.101]
6	2.291	2.361	[1.876, 2.835]	1.146	1.159	[0.877, 1.406]	−2.536	−2.524	[−3.068, −2.187]
7	2.131	2.185	[1.834, 2.576]	0.758	0.760	[0.595, 0.930]	1.782	1.759	[1.448, 2.081]
8	1.027	1.009	[0.806, 1.214]	1.720	1.736	[1.491, 2.009]	0.152	0.159	[−0.229, 0.225]
9	0.569	0.564	[0.403, 0.713]	1.119	1.152	[0.973, 1.324]	0.964	0.927	[0.735, 1.093]
10	0.578	0.550	[0.342, 0.761]	2.129	2.094	[1.776, 2.471]	1.462	1.485	[1.215, 1.745]
11	0.795	0.797	[0.615, 0.980]	1.445	1.466	[1.261, 1.691]	0.619	0.600	[0.376, 0.787]
12	2.279	2.389	[1.191, 2.867]	1.148	1.132	[0.875, 1.412]	−2.020	−2.028	[−2.388, −1.696]
13	0.714	0.616	[0.391, 0.864]	2.225	2.210	[1.867, 2.532]	0.602	0.577	[0.293, 0.826]
14	2.200	2.216	[1.797, 2.651]	1.465	1.471	[1.217, 1.721]	0.127	0.091	[−0.219, 0.381]
15	1.565	1.589	[1.349, 1.847]	0.728	0.711	[0.558, 0.867]	−0.587	−0.605	[−0.817, −0.419]
16	2.419	2.439	[2.076, 2.866]	2.408	2.380	[2.015, 2.796]	−0.218	−0.225	[−0.635, 0.094]
17	1.561	1.595	[1.342, 1.869]	1.398	1.388	[1.182, 1.621]	0.830	0.789	[0.533, 1.022]
18	2.457	2.470	[1.981, 2.900]	2.111	2.152	[1.792, 2.547]	1.558	1.560	[1.182, 1.926]
19	0.714	0.686	[0.545, 0.843]	0.918	0.883	[0.743, 1.030]	1.504	1.487	[1.320, 1.670]
20	2.447	2.482	[2.023, 2.942]	1.704	1.754	[1.490, 2.018]	0.126	0.110	[−0.221, 0.421]
21	1.588	1.562	[1.217, 1.905]	2.170	2.177	[1.825, 2.534]	−0.760	−0.789	[−1.123, −0.521]
22	1.724	1.721	[1.456, 2.037]	1.590	1.571	[1.320, 1.800]	0.769	0.671	[0.397, 0.912]
23	2.273	2.244	[1.909, 2.616]	0.948	0.917	[0.738, 1.119]	0.265	0.105	[−0.156, 0.343]
24	1.228	1.198	[0.902, 1.505]	2.782	2.755	[2.353, 3.128]	−1.398	−1.429	[−1.834, −1.115]
25	0.687	0.674	[0.456, 0.923]	2.261	2.275	[1.925, 2.651]	1.802	1.778	[1.429, 2.111]
26	1.665	1.666	[1.427, 1.928]	0.572	0.568	[0.443, 0.709]	0.033	0.021	[−0.172, 0.208]
27	2.383	2.400	[1.904, 2.823]	1.871	2.021	[1.626, 2.359]	1.307	1.285	[0.915, 1.620]
28	1.778	1.772	[1.443, 2.111]	2.326	2.305	[1.957, 2.641]	−0.871	−0.875	[−1.193, −0.581]
29	1.522	1.541	[1.175, 1.975]	2.909	2.934	[2.460, 3.505]	0.241	0.232	[−0.175, 0.588]
30	1.173	1.178	[1.940, 1.434]	1.703	1.710	[1.458, 1.977]	0.397	0.363	[0.104, 0.577]

* *indicates the constraints for model identification. True denotes the true value of parameter. EAP denotes the expected a priori estimation. HPDI denotes the 95% highest posterior density intervals*.

• Multilevel structural model at level 2 (individual level) can be represented by

(2.2) $$\begin{array}{l} \theta_{ijq}=\beta_{0jq}+x_{1ij}\beta_{1jq}+x_{2ij}\beta_{2jq}+\ldots +x_{hij}\beta_{hjq}+e_{ijq}, \end{array}$$

In Equation (2.2), the level-2 individual covariates are denoted as ***X***_*ij*_ = (*x*_1*ij*_, *x*_2*ij*_, …, *x*_*hij*_), where *h* is the number of individual background covariates. ***X***_*ij*_ can contain both continuous and discrete variables (e.g., socio-economic status, gender). The residual term, $e_{ij}={\left(e_{ij1},e_{ij2},\ldots ,e_{ijQ}\right)}^{\prime }$ is assumed to follow a multivariate normal distribution *N*(**0**, **Σ**_*e*_). Here, **Σ**_*e*_ is a *Q*-by-*Q* variance-covariance matrix. The individuals' abilities are considered to be the latent outcome variables of the multilevel regression model. Differences in abilities among individuals within the same school are modeled given student-level characteristics. Therefore, the explanatory information ***X***_*ij*_ at the individual level explains variability in the latent abilities within school.

• Level 3 (school level) model in this current study can be expressed as follows:

(2.3) $$\begin{array}{l} \beta_{hjq}=\gamma_{h0q}+w_{1j}\gamma_{h1q}+w_{2j}\gamma_{h2q}+\ldots +w_{sj}\gamma_{hsq}+u_{hjq}, \end{array}$$

In Equation (2.3), the level-3 school covariates are represented by $w_{j}={\left(w_{j1},w_{j2},\ldots ,w_{js}\right)}^{\prime }$, where *s* is the number of school covariates at level 3. Each level-2 random regression coefficient parameter is β_*hjq*_, which can be interpreted by school level covariates. The level-3 residual ${\left(u_{0jq},u_{1jq},\ldots u_{hjq}\right)}^{\prime }$ is multivariate normally distributed with mean **0** and (*h* + 1)-by-(*h* + 1) covariance matrix ***T***_*q*_, *q* = 1, …, *Q*. The variation across schools is modeled given background information at the school level. To control the model complexity, we assume that the level-3 residual covariance between different dimensions is 0; that is

(2.4) $$\begin{array}{l} Cov\left(u_{hjq_{1}},u_{hjq_{2}}\right)=0,\;q_{1},q_{2}\in 1,2,\ldots ,Q,\text{and }q_{1}\neq q_{2},\\ \;j=1,2,\ldots ,J,\;h=1,2,\ldots \end{array}$$

Different from Equation (2.2) in this paper, Huang and Wang (2014) proposed a high-order structure model to construct ability parameters with hierarchical strucutre. More specifically, all specific abilities linearly combine within a general ability. Assuming that there are two order of ability, including $\theta_{iqv}^{\left(1\right)}$ and $\theta_{iv}^{\left(2\right)}$, their relationship is described by the following model

(2.5) $$\begin{array}{l} \theta_{iqv}^{\left(1\right)}=\beta_{0qv}+\beta_{1qv}\theta_{iv}^{\left(2\right)}+\varepsilon_{iqv}^{\left(1\right)}, \end{array}$$

where $\theta_{iqv}^{\left(1\right)}$ and $\theta_{iv}^{\left(2\right)}$ denote first-order ability and second-order ability for the *i*th student sampled from school *v*, the subscript *q* denotes the dimension of the first-order ability. β_0*qv*_, β_1*qv*_, and $\varepsilon_{iqv}^{\left(1\right)}$ are the intercept, slope, and residual for the *q*th first-order ability in the *v*th school, respectively. $\varepsilon_{iqv}^{\left(1\right)}$ is the within-school residual and is typically assumed to be homogeneous across schools and normally distributed with a mean of zero and a variance of $\sigma_{\varepsilon }^{2}$ and independent of the other ***ε*** and ***θ***. However, in this current study, we only focus on the specific abilities of four dimensions without the general ability, which is the different between Huang and Wang (2014) and us in the construction of the ability structure model.

Moreover, in Huang and Wang (2014)'s paper, the multilevel data structure is investigated by introducing the individual level predictions directly into the above-mentioned higher-order ability model (Equation 2.5). The specific model is as follows:

(2.6) $$\begin{array}{l} \theta_{iqv}^{\left(1\right)}=\beta_{0qv}+\beta_{1qv}\theta_{iv}^{\left(2\right)}+\overset{H}{\underset{h=2}{\sum }}\beta_{hqv}G_{hiv}+\varepsilon_{iqv}^{\left(1\right)}, \end{array}$$

where *G*_*hiv*_ is the *h*th individual level predictor for the *i*th student in the *v*th school and β_*hqv*_ is its corresponding regression weight for the *q*th ability and school *v*. At the school level, the random coefficients ***β*** can be modeled as

(2.7) $$\begin{array}{l} \begin{array}{c} \beta_{0qv}=\gamma_{00q}+u_{0qv},\\ \beta_{1qv}=\gamma_{10q}+u_{1qv},\\ \beta_{hqv}=\gamma_{h0q}+u_{hqv}, \end{array} \end{array}$$

where *h* = 2, …, *H*, and the residuals $u_{v}\prime =(\mu_{0qv},\mu_{1qv},\ldots ,\mu_{Hqv})$ are assumed to follow a multivariate normal distribution with a mean vector of zero and a covariance matrix of Σ_*u*_. Further, school level predictors (e.g., school type, school size) can be added to the random intercept model. That is,

(2.8) $$\begin{array}{l} \beta_{0qv}=\gamma_{00q}+\overset{K}{\underset{k=1}{\sum }}\gamma_{kq}W_{kv}+u_{0qv}, \end{array}$$

where *W*_*kv*_ is the *k*th school level predictor and γ_*kv*_ is its corresponding regression weight for the *q*th ability.

However, in this current study, the multiple dimensional abilities are directly built into the random regression models through the individual level predictors (Equation 2.2). It is not the same as Huang and Wang (2014, p. 498, Equation 4) that constructs hierarchical structure ability and multilevel data in one model. In addition, when constructing the school level models in our paper, school level predictive variables, such as teacher satisfaction, school climate, are used to model the random intercept and random slopes (Equation 2.3). Considering if different predictors are added to the school level model, multiple versions of the school level models are generated. Therefore, we can use the Bayesian model assessment to select the best-fitting model. However, Huang and Wang (2014) only model the random intercept by predictive variables at school level, without considering the impact of predictive variables on other random coefficients (page 498, Equation 8).

## 3. Bayesian Parameter Estimation and Model Selection

### 3.1. Identifying Restrictions

In this current study, the multilevel multidimensional IRT models are identified based on discrimination and difficulty parameters (Fraser, 1988; Béguin and Glas, 2001; Skrondal and Rabe-Hesketh, 2004). The most convenient method is to set *Q* item parameters *b*_*k*_ equal to 0 if *k* = *q*, and impose the restrictions *a*_*kq*_ = 1, where *k* = 1, 2, …*Q*, and *q* = 1, …, *Q*. If *k* ≠ *q*, *a*_*kq*_ = 0. If *k* > *q*, *b*_*k*_ and *a*_*kq*_ will be free parameters to estimate. The basic idea is to identify the model by anchoring several item discrimination parameters to an arbitrary constant, typically *a*_*kq*_ = 1. Meanwhile, the location identification constrains is required by restricting the difficulty parameters for given items, typically, *b*_*k*_ = 0. Based on the fixed anchoring values of item parameters, other parameters are estimated on the same scale. The estimated difficulty or discrimination values of item parameters are interpreted based on their relative positions to the corresponding anchoring values (Béguin and Glas, 2001, p. 545). Additionally, in order to have a clear understanding of the process of restricting the identifiability, we illustrate the identifiability of the two-dimensional models. For details, please refer to item 1 and item 2 in Tables 1, 2 for the restrictions of discrimination and difficult parameters.

**Table 2:** Parameter estimates of the fixed effect, Level-2 variance-covariance and Level-3 variance-covariance in simulation 1.

**Fixed effect**	**True**	**EAP**	**HPDI**	**Fixed effect**	**True**	**EAP**	**HPDI**
γ_001_	1.000	0.982	[0.928, 1.225]	γ_002_	−0.350	−0.377	[−0.659, −0.115]
γ_011_	0.300	0.326	[0.129, 0.510]	γ_012_	0.300	0.281	[−0.046, 0.524]
γ_101_	0.500	0.521	[0.244, 0.807]	γ_102_	0.500	0.522	[0.296, 0.824]
γ_111_	0.350	0.325	[0.134, 0.501]	γ_112_	−1.000	−0.986	[−1.234, −0.736]
**Level-2 random effect**			**True**	**EAP**		**HPDI**	
$\sigma_{e_{1}}^{2}$			0.300	0.323		[0.269, 0.387]	
σ_*e*_1_*e*_2__			0.075	0.093		[0.053, 0.136]	
σ_*e*_2_*e*_1__			0.075	0.093		[0.053, 0.136]	
$\sigma_{e_{2}}^{2}$			0.500	0.529		[0.438, 0.648]	
**Level-3** ***T*_1_**	**True**	**EAP**	**HPDI**	**Level-3** ***T*_2_**	**True**	**EAP**	**HPDI**
τ_001_	0.100	0.115	[0.016, 0.380]	τ_002_	0.100	0.073	[−0.058, 0.369]
τ_011_	0	0.013	[−0.229, 0.140]	τ_012_	0	0.017	[−0.143, 0.192]
τ_101_	0	0.013	[−0.229, 0.140]	τ_102_	0	0.017	[−0.143, 0.192]
τ_111_	0.100	0.074	[−0.068, 0.436]	τ_112_	0.100	0.119	[−0.093, 0.298]

### 3.2. Gibbs Sampling Within the MCMC Framework

In the framework of frequentist, two commonly used estimation methods are used to estimate the complex IRT models. One is the marginal maximum likelihood estimation (MMLE; Bock and Aitkin, 1981), and the other is the weighted least squares means and variance adjusted (WLSMV; Muthén et al., 1997). However, the main disadvantage of the marginal maximum likelihood method is that it inevitably needs to approximate the tedious multidimensional integral by using numerical or Monte Carlo integration, which will increase large the computational burden. Another disadvantage of the MMLE are that it is difficulty to incorporate uncertainty (standard errors) into parameter estimates (Patz and Junker, 1999a), and the comparison method of the MMLE is simplistic, except the RMSEA (Root Mean Square Error of Approximation) which is often used, other comparison methods are seldom used. In addition, there are some disadvantages in WLSMV compared with Bayesian method used in this paper. Firstly, Bayesian method outperforms WLSMV solely in case of strongly informative accurate priors for categorical data. Even if the weakly informative inaccurate priors are used when the sample size is moderate and not too small, the performance of Bayesian method does not deteriorate (Holtmann et al., 2016). Secondly, compared with WLSMV, Bayesian method does not rely on asymptotic arguments and can give more reliable results for small samples (Song and Lee, 2012). Thirdly, Bayesian method allows the possibility to analyze models that are computationally heavy or impossible to estimate with WLSMV (Asparouhov and Muthén, 2012). For example, the computational burden of the WLSMV becomes intensive especially when a large number of items is considered. Fourth, Bayesian method has a better convergence rate compared with WLSMV. Fifth, Bayesian method can be used to evaluate the plausibility of the model or its general assumptions by using posterior predictive checks (PPC; Gelman et al., 1996). For the above-mentioned reasons, Bayesian method is chosen for estimating the following multilevel multidimensional IRT models.

In fact, Bayesian methods have been widely applied to estimate parameters in complex multilevel IRT models (e.g., Albert, 1992; Bradlow et al., 1999; Patz and Junker, 1999a,b; Béguin and Glas, 2001; Rupp et al., 2004). Within the framework of Bayesian, a series of BUGS softwares can be used to estimate these multilevel IRT models, including OpenBUGS (Spiegelhalter et al., 2003) and JAGS (Plummer, 2003). However, in this paper, we implement the Gibbs sampling by introducing the augmented variables rather than by constructing an envelope of the log of the target density as in a series of BUGS softwares. The auxiliary or latent variable approach has several important advantages. First, the approach is very flexible and can handle almost all sorts of discrete responses. Typically, the likelihood of the observed response data has a complex structure but the likelihood of the augmented (latent) data has a known distribution with convenient mathematical properties. Second, conjugate priors, where the posterior has the same algebraic form as the prior, can be more easily defined for the likelihood of the latent response data, which has a known distributional form, than for the likelihood of the observed data. Third, the augmented variable approach facilitates easy formulation of a Gibbs sampling algorithm based on data augmentation. It will turn out that by augmenting with a latent continuous variable, conditional distributions can be defined based on augmented data, from which samples are easily drawn. Fourth, the conditional posterior given augmented data has a known distributional form such that conditional probability statements can be directly evaluated for making posterior inferences. The likelihood of the augmented response data is much more easily evaluated than the likelihood of the observed data and can be used to compare models. In summary, in this study, we adopt the Gibbs sampling algorithm (Geman and Geman, 1984) with data augmentation (Tanner and Wong, 1987) to estimate multilevel multidimensional IRT models. In particular, let ***θ*** and **ξ** denote the vectors of all person and item parameters. Define an augmented variable *Z*_*ijk*_ that is normally distributed with mean $\eta_{ijk}=\overset{Q}{\underset{q=1}{\sum }}a_{kq}\theta_{ijq}-b_{k}$ and variance 1.

The joint posterior distribution of the parameters given the data is as follows:

(3.1) $$\begin{array}{l} p\left(Z,\;\theta ,\;\xi ,\;\beta ,\Sigma_{e},\;\gamma ,\;T|Y,\;X,\;W\right)\propto \overset{n_{j}}{\underset{i=1}{\prod }}\overset{J}{\underset{j=1}{\prod }}\overset{K}{\underset{k=1}{\prod }}\overset{Q}{\underset{q=1}{\prod }}\\ p\left(Z_{ijk}|\theta_{ijq},\;\xi_{k},Y_{ijk}\right)p\left(\theta_{ijq}|\beta_{jq},\sigma_{q}^{2},\;X_{j}\right)\\ \times p\left(\beta_{jq}|\gamma_{q},\;T_{q},\;W_{j}\right)p\left(\gamma_{q}|T_{q}\right)p\left(\xi_{k}\right)p\left(\Sigma_{e}\right)p\left(T_{q}\right). \end{array}$$

where $\sigma_{q}^{2}$ is the conditional variance given the other ability dimensions. It can be obtained from **Σ**_*e*_. The details of the Gibbs sampling are shown as follows

**Step 1**: Sampling ***Z*** given the parameters ***θ*** and **ξ**, where the random variable *Z*_*ijk*_ is independent

(3.2) $$Z_{ijk}|\theta ,\xi ,Y\sim \{\begin{array}{l} N\left(\overset{Q}{\underset{q=1}{\sum }}a_{kq}\theta_{ijq}-b_{k},1\right)\text{ truncated at the left by 0 if }Y_{ijk}=1,\\ N\left(\overset{Q}{\underset{q=1}{\sum }}a_{kq}\theta_{ijq}-b_{k},1\right)\text{ truncated at the right by 0 if }Y_{ijk}=0. \end{array}$$

**Step 2**: Sampling ***θ***_*ij*_ according to Gibbs sampling characteristics. A divide-and-conqueror strategy is used to draw each sampling element of $\theta_{ij}={\left(\theta_{ij1},\;\theta_{ij\left(-1\right)}\right)}^{\prime },$ where ***θ***_*ij*(−1)_ = (θ_*ij*2_, ···, θ_*ijQ*_). Let $\beta_{j}={\left(\beta_{j1},\cdot \cdot \cdot ,\beta_{jQ}\right)}^{\prime },$ $\mu =\left(X_{ij}\beta_{j1},\;\mu_{1}^{\left(2\right)}\right)\prime ,$ where $\mu_{1}^{\left(2\right)}=\left(X_{ij}\beta_{j2},\cdot \cdot \cdot ,X_{ij}\beta_{jQ}\right)$ and $\Sigma_{e}=\left(\begin{array}{cc} \sigma_{e_{1}}^{2} & \Sigma_{12}\\ \Sigma_{21} & \Sigma_{22} \end{array}\;\right).$ The conditional prior distribution of θ_*ij*1_ can be written as

$$p\left(\theta_{ij1}|\theta_{ij\left(-1\right)},\;\beta_{j},\Sigma_{e}\right)\sim N\left(\mu_{ij}^{1},\;\sigma_{1}^{2}\right),$$

$$\mu_{ij}^{1}=X_{ij}\beta_{j1}+\Sigma_{12}\Sigma_{22}^{-1}\left(\theta_{ij\left(-1\right)}-\mu_{1}^{\left(2\right)}\right),\;\sigma_{1}^{2}=\sigma_{e_{1}}^{2}-\Sigma_{12}\Sigma_{22}^{-1}\Sigma_{21}.$$

Therefore, the full conditional posterior density of θ_*ij*1_ (Lindley and Smith, 1972; Box and Tiao, 1973) is given by

(3.3) $$\begin{array}{r} \theta_{ij1}|Z_{ij},\theta_{ij(-1)},\xi ,\beta_{j1},\sigma_{1}^{2}\sim N({\left(v+\sigma_{1}^{2}\right)}^{-1}({\tilde{\theta }}_{ij1}\sigma_{1}^{2}+\mu_{ij}^{1}v),\\ {\left(v+\sigma_{1}^{2}\right)}^{-1}(v\sigma_{1}^{2})).\; \end{array}$$

where

$${\tilde{\theta }}_{ij1}={\left(\sum_{k=1}^{K}a_{k1}^{2}\right)}^{-1}\left[\sum_{k=1}^{K}a_{k1}(Z_{ijk}+b_{k}-a_{k2}\theta_{ij2}-\cdot \cdot \cdot -a_{kQ}\theta_{ijQ})\right],$$

$v={\left(\overset{K}{\underset{k=1}{\sum }}a_{k1}^{2}\right)}^{-1}.$ For *q* = 2, …, *Q*, θ_*ijq*_ can be drawn in the same manner.

**Step 3:** Sampling ***ξ***_*k*_, $\xi_{k}={\left(a_{k1},\cdot \cdot \cdot ,a_{kQ},b_{k}\right)}^{\prime },$ Given $\theta ,Z_{k}={\left(Z_{11k},\cdot \cdot \cdot ,Z_{n_{1}1k},\cdot \cdot \cdot ,Z_{n_{J}Jk}\right)}^{\prime },$ Here *n* (*n* = *n*_1_ + *n*_2_ + ··· + *n*_*J*_) represents the total number of individuals in different groups. The residual can be written as ***ε***_*k*_ $={\left(\varepsilon_{11k},\cdot \cdot \cdot ,\varepsilon_{n_{1}1k},\cdot \cdot \cdot ,\varepsilon_{n_{J}Jk}\right)}^{\prime }$ and each element is distributed as *N*(0, 1). Therefore, we have

$$Z_{k}=\left[\theta \;-1\right]\xi_{k}+\varepsilon_{k}.$$

Let ***H*** = [***θ*** − 1], the likelihood function of ***ξ***_*k*_ is normally distributed with mean ${\overset{˜}{\xi }}_{k}={\left(H\prime \;H\right)}^{-1}H\prime \;Z_{k}$ and $H_{0}={\left(H\prime \;H\right)}^{-1}$. Suppose that the priors of the discrimination and difficult parameters are ***a***_*k*_ ~ *N*(***μ***_*a*_, Σ_*a*_) I (***a***_*k*_|*a*_*kq*_ > 0, *q* = 1, …, *Q*) and $b_{k}\sim N\left(\mu_{b},\sigma_{b}^{2}\right)$, respective, Here $\mu_{a}={\left(\mu_{a1},\ldots ,\mu_{aQ}\right)}^{\prime }$ and $\Sigma_{a}=diag\left(\sigma_{a1}^{2},\ldots ,\sigma_{aQ}^{2}\right)$. The prior of item parameter ***ξ***_*k*_ is a multivariate normal distribution with mean $\mu_{\xi_{0}}={\left(\mu_{a1},\ldots ,\mu_{aQ},\mu_{b}\right)}^{\prime }$and $\Sigma_{\xi_{0}}=diag\left(\sigma_{a1}^{2},\ldots ,\sigma_{aQ}^{2},\sigma_{b}^{2}\right)$. Therefore, the full conditional posterior distribution of the item parameters is given by

(3.4) $$\begin{array}{l} \xi_{k}|\theta ,Z_{k},Y\sim N({\left(H_{0}^{-1}+\Sigma_{\xi_{0}}^{-1}\right)}^{-1}(H\prime \;Z_{k}+\Sigma_{\xi_{0}}^{-1}\mu_{\xi_{0}}),\\ \;{\left(H_{0}^{-1}+\Sigma_{\xi_{0}}^{-1}\right)}^{-1})\text{I}(a_{k}|a_{kq}>0,q=1,\ldots ,Q). \end{array}$$

**Step 4:** Sampling ***β***_*j*_=${\left(\beta_{j1},\ldots ,\beta_{jQ}\right)}^{\prime },$ given ***θ***, $\sigma_{q}^{2},$ ***γ*** and ***T***. Dawn an element of vector ***β***_*j*_, $\beta_{j1}={\left(\beta_{0j1},\ldots ,\beta_{hj1}\right)}^{\prime }.$ Let $\theta_{j1}={\left(\theta_{1j1},\ldots ,\theta_{n_{j}j1}\right)}^{\prime },$ and ${\text{X}}_{j}={\left({\text{X}}_{1j},\ldots ,{\text{X}}_{n_{j}j}\right)}^{\prime },$ with ***X***_*ij*_ as defined in the part of model introduction. The level-2 residual ***e***_*j*1_ can be defined as $e_{j1}={\left(e_{1j1},\ldots ,e_{n_{j}j1}\right)}^{\prime }.$ Therefore, we have

$$\theta_{j1}=X_{j}\beta_{j1}+e_{j1}.$$

The level-2 likelihood function of ***β***_*j*1_ is normally distributed with mean ${\tilde{\beta }}_{j1}={\left(X_{j}^{\prime }X_{j}\right)}^{-1}X_{j}^{\prime }\theta_{j1}$ and variance $\Sigma_{j1}=\sigma_{1}^{2}{\left(X_{j}^{\prime }X_{j}\right)}^{-1}$. Furthermore, ***w***_*j*_ is the direct product of ***w***_*js*_ = (1, *w*_*j*1_, …, *w*_*js*_) and a (*h* + 1) identity matrix, that is, ***w***_*j*_ = ***I***_(*h* + 1)_ ⊗ ***w***_*js*_. The random regression coefficient ***β***_*j*1_ is induced by a normal prior at level 3 with mean ***w***_*j*_***γ***_1_ and covariance ***T***_1_, where $\gamma_{1}={\left(\gamma_{001},\gamma_{011}\ldots ,\gamma_{0s1},\ldots ,\gamma_{h01},\gamma_{h11},\ldots ,\gamma_{hs1}\right)}^{\prime }.$ The level-3 residual ***u***_*j*1_ can be defined as $u_{j1}={\left(u_{0j1},\ldots ,u_{hj1}\right)}^{\prime }.$ Therefore, we have

$$\beta_{j1}=w_{j}\gamma_{1}+u_{j1}.$$

Thus, the fully conditional posterior distribution of ***β***_*j*1_ is given by

(3.5) $$\begin{array}{l} \beta_{j1}|\theta_{j1},\sigma_{1}^{2},\gamma_{1},T_{1}\sim N({\left(\Sigma_{j1}^{-1}+T_{1}^{-1}\right)}^{-1}\\ \;(\Sigma_{j1}^{-1}{\tilde{\beta }}_{j1}+T_{1}^{-1}w_{j}\gamma_{1}),\;{\left(\Sigma_{j1}^{-1}+T_{1}^{-1}\right)}^{-1}), \end{array}$$

and ***β***_*jq*_, *q* = 2, …, *Q*, is drawn in the same manner.

**Step 5:** Sampling ***γ***, ***γ*** = (***γ***_1_, ···, ***γ***_*Q*_). An element of vector ***γ*** is drawn, and the matrix ***γ***_1_ is the matrix of regression coefficients corresponding to the regression of ***β***_*j*1_ on ***w***_*j*_. An improper noninformative prior density for ***γ***_1_ is used. Similar prior is used as shown in Fox and Glas (2001). Therefore, the full conditional posterior distribution of ***γ***_1_ is given by

(3.6) $$\begin{array}{l} \gamma_{1}|\beta_{j1},\;T_{1}\sim N\left({\left(\overset{J}{\underset{j=1}{\sum }}w_{j}^{\prime }T_{1}^{-1}w_{j}\right)}^{-1}\overset{J}{\underset{j=1}{\sum }}w_{j}^{\prime }T_{1}^{-1}\beta_{j1},\;{\left(\overset{J}{\underset{j=1}{\sum }}w_{j}^{\prime }T_{1}^{-1}w_{j}\right)}^{-1}\right), \end{array}$$

and ***γ***_*q*_ is drawn in the same manner for *q* = 2, ···, *Q*.

**Step 6:** Sampling the residual variance-covariance structure Σ_*e*_. A prior for Σ_*e*_ is an Inverse-Wishart$\left(v_{0},\Sigma_{0}^{-1}\right)$ distribution. The full conditional posterior distribution of Σ_*e*_ is given by

(3.7) $$\begin{array}{l} \Sigma_{e}|\theta ,\;\beta \sim \text{Inverse-Wishart }\;(v_{0}+N,{\left(S+\Sigma_{0}\right)}^{-1}) \end{array}$$

where $S=\overset{J}{\underset{j=1}{\sum }}\overset{n_{j}}{\underset{i=1}{\sum }}\left(\theta_{ij}-{\text{X}}_{ij}\beta_{j}\right){\left(\theta_{ij}-{\text{X}}_{ij}\beta_{j}\right)}^{\prime },$where *N* = *J* × *n*_*j*_.

**Step 7:** Sampling the level-3 variance-covariance structure ***T*** = *diag* (***T***_1_, ···, ***T***_*Q*_). ***T***_1_ is drawn first. A prior for ***T***_1_ is an Inverse-Wishart$\left(v_{1},\Sigma_{1}^{-1}\right)$ distribution. The full conditional posterior distribution of ***T***_1_ is given by

(3.8) $$\begin{array}{l} T_{1}|\beta_{j1},\;\gamma_{1}\sim \text{Inverse-Wishart }\left(v_{1}+J,{\left(S_{1}+\Sigma_{1}\right)}^{-1}\right) \end{array}$$

where $S_{1}=\overset{J}{\underset{j=1}{\sum }}\left(\beta_{j1}-w_{j}\gamma_{1}\right){\left(\beta_{j1}-w_{j}\gamma_{1}\right)}^{\prime },$and ***T***_*q*_ is drawn in the same manner for *q* = 2, ···, *Q*.

### 3.3. Model Selection

The deviance information criterion (DIC) was introduced by Spiegelhalter et al. (2002) as a model selection criterion for the Bayesian hierarchical models. Similar to many other criteria (such as the Bayesian information criterion or BIC; BIC is not intended to predict out-of-sample model performance but rather is designed for other purposes, we do not consider it further here (Gelman et al., 2014), it trades a measure of model adequacy against a measure of complexity. Specifically, the DIC is defined as the sum of a deviance measure and a penalty term for the effective number of parameters based on a measure of model complexity. The model with a larger DIC has a better fit to the data. In the framework of a multilevel IRT models, the performances of DICs based on five versions of deviances have been investigated in Zhang et al. (2019). The DIC used in this current study belongs to the top-level marginalized DIC in their paper. The reason for using the top-level marginalized DIC in our paper is that our main purpose is to investigate the influences of fixed effects (***γ***) on the multiple dimensional abilities. Therefore, the deviance is defined at the highest level fixed effects (***γ***), where the random effects of intermediate processes, such as the second-level random individual ability effects ***θ*** or the third-level random coefficient effects ***β***, will not be considered in the defined deviance. Next, the calculation formula of the top-level marginalized DIC is given.

Let **Ω**_1_ = (***ξ***, **Σ**_*e*_, ***T***) (**Ω**_1_ do not include the intermediate process random parameters ***θ*** and ***β***). According to the augmented data likelihood *p*(***Z***|**Ω**_1_), we can obtain the following deviance

$$D\left(\gamma \right)=-2\log p\left(Z|\Omega_{1}\right).$$

Then the top-level marginalized DIC is defined as

(3.9) $$\begin{array}{l} \text{DIC}=\int \left[\text{DIC}|Z,\;\Omega_{1}\right]\cdot p\left(Z,\;\Omega_{1}|Y\right)dZd\Omega_{1}\\ \;=\int \left[D\left(\bar{\gamma }|Z,\;\Omega_{1}\right)+2p_{D}\left(Z,\;\Omega_{1}\right)\right]\cdot p\left(Z,\;\Omega_{1}|Y\right)dZd\Omega_{1}\\ \;=E_{Z,\;\Omega_{1}}\left[D\left(\bar{\gamma }\right)+2p_{D}\left(Z,\;\Omega_{1}\right)|Y\right] \end{array}$$

In Equation (3.9), the conditional DIC is a function of ***Z*** and **Ω**_1_, which can be written as [DIC|**Z**, **Ω**_1_]. $D\left(\bar{\gamma }\right)$ denotes the deviance of the posterior estimation mean given augmented data ***Z*** and **Ω**_1_. *p*_*D*_(**Z**, **Ω**_1_) is the effective number of parameters given the augmented data ***Z*** and **Ω**_1_, which can be expressed as $p_{D}\left(\text{Z},\;\Omega_{1}\right)=\bar{D\left(\gamma \right)}-D\left(\bar{\gamma }\right)$.

An important advantage of DIC is that it can be easily calculated from the generated samples. It can be obtained by MCMC sampling augmentation auxiliary variable ***Z*** and structural parameters **Ω**_1_ from the joint posterior distribution *p*(**Z**, **Ω**_1_|**Y**).

## 4. Simulation

### 4.1. Simulation 1

A simulation study is conducted to evaluate the performance of the proposed Gibbs sampler MCMC method for recovering the parameters of the multilevel IRT models. For illustration purposes, we only consider one explanatory variable on both levels, and the number of dimensions is fixed at 2 (*q* = 2). The true structural multilevel model is simplified as

The individual-level model:

(4.1) $$\begin{array}{l} \theta_{ijq}=\beta_{0jq}+x_{ij}\beta_{1jq}+e_{ijq}, \end{array}$$

where

(4.2) $$\begin{array}{l} e=\left(\begin{array}{c} e_{ij1}\\ e_{ij2} \end{array}\right)\sim N\left(\left(\begin{array}{c} 0\\ 0 \end{array}\;\right),\left(\begin{array}{cc} \sigma_{e_{1}}^{2} & \sigma_{e_{1}e_{2}}\\ \sigma_{e_{2}e_{1}} & \sigma_{e_{2}}^{2} \end{array}\right)\right). \end{array}$$

The school-level model:

(4.3) $$\begin{array}{l} \beta_{0jq}=\gamma_{00q}+\gamma_{01q}w_{j}+u_{0jq},\\ \beta_{1jq}=\gamma_{10q}+\gamma_{11q}w_{j}+u_{1jq}, \end{array}$$

where

(4.4) $$\begin{array}{l} \left(\begin{array}{c} u_{0jq}\\ u_{1jq} \end{array}\;\right)\sim N\left(\left(\begin{array}{c} 0\\ 0 \end{array}\right),\;T\right),\;T=\left(\begin{array}{cc} \tau_{00q} & \tau_{01q}\\ \tau_{10q} & \tau_{11q} \end{array}\;\right). \end{array}$$

We use the multidimensional two-parameter normal ogive model to generate the responses. The test length is set to 30. In the multidimensional item response theory book, Reckase (2009, p. 93) points out that the each element of discrimination parameter vectors, *a*_*kq*_, can take on any values except the usual monotonicity constraint that requires the values of the elements of ***a***_*k*_ be positive, where $a_{k}={\left(a_{k1},a_{k2}\right)}^{\prime }$. Therefore, we adopt the truncated normal distribution with mean 1.5 and variance 1 to generate the true value of the each element of discrimination parameter vectors ***a***_*k*_. That is, *a*_*kq*_ ~ *N*(1.5, 1) *I* (*a*_*kq*_ > 0), *q* = 1, 2, *k* = 1, …, 30. For the difficulty parameter, the selection of the true values is the same as that of the traditional unidimensional IRT models. Here we assume that the difficult parameters are generated from the standard normal distribution. That is, *b*_*k*_ ~ *N*(0, 1), *k* = 1, …, 30. The ability parameters of 2,000 students from population *N*(**X**_*ij*_***β***_*j*_, **Σ**_*e*_) are divided into *J* = 10 groups, with *n*_*j*_(200) students in each group. The fixed effect ***γ*** is chosen as an arbitrary value between −1 and 1. For simplicity, we suppose that at level 3, each of the dimensional covariances τ_01*q*_ and τ_10*q*_ is equal to 0 for *q* = 1, 2, which means that the level-3 residuals between random coefficients ***β***_*q*_ = (β_0*jq*_, β_01*jq*_) are independent of each other. The level-3 variances τ_00*q*_ and τ_11*q*_ are, respectively, set equal to 0.100, for *q* = 1, 2 such that they have very low stochastic volatility in the vicinity of the level-3 mean. The level-2 residual variance-covariance (VC) are set to 0.300, 0.500, and 0.075. The explanatory variables ***X*** and **W** are drawn from *N* (0.25, 1) and *N* (0.5, 1), respectively.

The posterior distribution in the Bayesian framework can be obtained by connecting with the likelihood function (sample information) and prior distribution (prior information). In general, the two kinds of information have important influence on the posterior distribution. In large scale educational assessment, the number of examinees is often very large, for example, in our real data study, the number of examinees and items, respectively, reach 2000 and 124. Therefore, the likelihood information plays a dominant role, and the selection of different priors (informative or non-informative) has no significant influence on the posterior inferences. As a result, the non-informative priors are often used in many educational measurement studies, e.g., van der Linden (2007) and Wang et al. (2018). In this paper, the prior specification will be uninformative enough for the data to dominate the prior, so that the influence of the prior on the results will be minimal. Next, we give the prior distributions of parameters involved in the simulation 1. The priors of the discrimination parameters and difficulty parameters are set as the non-informative priors $a_{k}\sim N\left(\left(\begin{array}{l} 0\\ 0 \end{array}\right),\left(\begin{array}{ll} 100 & 0\\ 0 & 100 \end{array}\right)\right)\text{I}(a_{k}|a_{k1}>0,a_{k2}>0)$ and *N* (0, 100). The fixed effect ***γ*** follows a uniform distribution *U* (−2, 2). The prior to the VC matrix of the level-2 ability dimensions is a 2-by-2 identity matrix. As used in many educational and psychological research studies (see Fox and Glas, 2001; Kim, 2001; Sheng, 2010), the priors to the VC matrices of the level-3, ***T***_1_ and ***T***_2_, are set to the non-informative priors based on Fox and Glas (2001)'s paper (see Fox and Glas, 2001), where *p* (***T***_*q*_) ∝ 1, *q* = 1, 2.

The convergence of Gibbs sampler is checked by monitoring the trace plots of the parameters for consecutive sequences of 20,000 iterations. The trace plots of two items randomly selected, fixed-effect parameters, level-2 residual variance-covariance component parameters and level-3 residual variance-covariance component parameters are shown in Supplementary Material. The trace plots show that all parameter estimates stabilize after 5,000 iterations and then converge quickly. Thus, we set the first 5,000 iterations as the burn-in period. In addition, the Brook-Gelman ratio diagnostic Brooks and Gelman (1998) ($\hat{R}$; as updated Gelman-Rubin statistic) plots are used to monitor the convergence and stability. Four chains started at overdispersed starting values are run for monitoring the convergence. Our Brook-Gelman ratios are close to 1.2. The true values, the expected a priori (EAP) estimation and the 95% highest posterior density intervals (HPDIs) for item parameters are shown in Table 1. Table 2 presents the true values and the estimated values of fixed effects ***γ***, level-2 covariance components, and level-3 variance components ***T***_1_ and ***T***_2_.

The accuracy of the parameter estimates is measured by two evaluation indexes, namely, Bias and root mean squared error (RMSE). The recovery results are based on 100 times MCMC repeated iterations. That is, 100 replicas are generated. The results of the accuracy of the parameter estimates are displayed in Tables 3, 4. From Tables 3, 4, we see that Gibbs sampling algorithm provides accurate estimates of the item parameters and multilevel structure parameters in the sense of having small Bias and RMSE values.

**Table 3:** Evaluating the accuracy of item parameter estimation.

	***a***_***k*1**_			***a***_***k*2**_			***b***_***k***_		
**Item**	**True**	**Bias**	**RMSE**	**True**	**Bias**	**RMSE**	**True**	**Bias**	**RMSE**
1	1^*^	0	0	0^*^	0	0	0^*^	0	0
2	0^*^	0	0	1^*^	0	0	0^*^	0	0
3	0.914	−0.037	0.114	0.686	−0.014	0.090	−1.182	0.028	0.144
4	1.102	0.025	0.098	1.468	0.017	0.125	0.441	−0.015	0.093
5	2.055	−0.010	0.073	1.428	0.025	0.047	−1.197	−0.170	0.137
6	2.291	0.070	0.153	1.146	0.013	0.084	−2.536	0.012	0.126
7	2.131	0.054	0.119	0.758	0.002	0.035	1.782	−0.023	0.149
8	1.027	−0.018	0.159	1.720	0.016	0.140	0.152	0.007	0.094
9	0.569	−0.005	0.136	1.119	0.033	0.102	0.964	−0.037	0.072
10	0.578	−0.019	0.180	2.129	−0.035	0.185	1.462	0.023	0.103
11	0.795	0.002	0.088	1.445	0.021	0.137	0.619	−0.019	0.081
12	2.279	0.110	0.153	1.148	−0.016	0.098	−2.020	−0.008	0.053
13	0.714	−0.098	0.142	2.225	−0.015	0.053	0.602	−0.025	0.091
14	2.200	0.016	0.093	1.465	0.006	0.039	0.127	0.036	0.127
15	1.565	0.024	0.120	0.728	−0.017	0.092	−0.587	−0.018	0.116
16	2.419	0.020	0.162	2.408	−0.028	0.164	−0.218	−0.007	0.092
17	1.561	0.034	0.105	1.398	−0.010	0.072	0.830	−0.041	0.115
18	2.457	0.013	0.091	2.111	0.041	0.109	1.558	0.002	0.150
19	0.714	−0.028	0.155	0.918	−0.035	0.156	1.504	−0.017	0.197
20	2.447	0.035	0.198	1.704	0.050	0.143	0.126	−0.016	0.156
21	1.588	−0.026	0.185	2.170	0.007	0.124	−0.760	0.029	0.256
22	1.724	−0.003	0.147	1.590	−0.019	0.128	0.769	−0.098	0.153
23	2.273	−0.029	0.084	0.948	−0.031	0.060	0.265	−0.160	0.179
24	1.228	−0.030	0.189	2.782	−0.027	0.194	−1.398	−0.031	0.132
25	0.687	−0.013	0.075	2.261	0.014	0.107	1.802	0.024	0.193
26	1.665	0.001	0.120	0.572	−0.004	0.068	0.033	−0.012	0.090
27	2.383	0.017	0.148	1.871	0.015	0.095	1.307	0.022	0.158
28	1.778	−0.008	0.113	2.326	−0.021	0.140	−0.871	−0.004	0.083
29	1.522	0.019	0.096	2.909	0.025	0.163	0.241	0.009	0.127
30	1.173	0.005	0.181	1.703	0.007	0.098	0.397	−0.034	0.221

* *indicates the constraints for model identification. RMSE denotes the root mean squared error*.

**Table 4:** Evaluating the accuracy of the two-dimensional fixed effects and variance-covariance components.

**Fixed effect**	**True**	**Bias**	**RMSE**	**Fixed effect**	**True**	**Bias**	**RMSE**
γ_001_	1.000	−0.018	0.082	γ_002_	−0.350	−0.027	0.169
γ_011_	0.300	0.026	0.156	γ_012_	0.300	−0.019	0.096
γ_101_	0.500	0.021	0.148	γ_102_	0.500	0.022	0.147
γ_111_	0.350	−0.025	0.173	γ_112_	−1.000	0.014	0.121
**Level-2 random effect**			**True**	**Bias**		**RMSE**	
$\sigma_{e_{1}}^{2}$			0.300	0.023		0.098	
σ_*e*_1_*e*_2__			0.075	0.018		0.163	
σ_*e*_2_*e*_1__			0.075	0.018		0.163	
$\sigma_{e_{2}}^{2}$			0.500	0.029		0.117	
**Level-3** ***T*_1_**	**True**	**Bias**	**RMSE**	**Level-3** ***T*_2_**	**True**	**Bias**	**RMSE**
τ_001_	0.100	0.015	0.164	τ_002_	0.100	−0.029	0.143
τ_011_	0	0.013	0.182	τ_012_	0	0.017	0.187
τ_101_	0	0.013	0.182	τ_102_	0	0.017	0.187
τ_111_	0.100	−0.026	0.139	τ_112_	0.100	0.019	0.167

### 4.2. Simulation 2

The purpose of this simulation study is to verify whether the Gibbs sampling algorithm can guarantee the accuracy of parameters estimation when the dimensions of latent ability increase so that it can be used to guide real data analysis later. The simulation design is as follows.

The number of dimensions is fixed at 4. The multidimensional normal ogive IRT model is used to generate responses. Two factors and their varied conditions are considered: (a) number of individuals, *N* = 1,000, 2,000, or 3,000; (b) number of items, *K* = 40, 100, or 200, and for per subtest number of itmes, 10, 25, or 50. Fully crossing the different levels of these two factors yield 9 conditions. Individuals (*N* = 1,000, 2,000, 3,000) are equally distributed to 10 schools (*J* = 10). True values of item parameters and priors of all of parameters are generated by the same in simulation 1. The true values of the fixed effects are, respectively, 1.000(γ_00*q*_), 0.300(γ_01*q*_), 0.500(γ_10*q*_) and 0.350(γ_11*q*_), *q* = 1, 2, 3, 4, and the level-2 variance are 0.300$\left(\sigma_{e_{1}}^{2}\right)$, 0.500$\left(\sigma_{e_{2}}^{2}\right)$, 0.750$\left(\sigma_{e_{3}}^{2}\right)$, and 1.000$\left(\sigma_{e_{4}}^{2}\right),$ and the covariance are set to 0.075. The level-3 variance are 0.1 (τ_00*q*_, τ_11*q*_), and the covariance are 0 (τ_01*q*_, τ_10*q*_). The multilevel structural models (Equations 2.2 and 2.3) in simulation study 1 are used, but the dimensions are fixed at 4.

The accuracy of the parameter estimates is measured by two evaluation indexes, namely, Bias and RMSE. The recovery results are based on the MCMC iterations repeated 100 times. The detail results of the accuracy of the parameter estimates under nine conditions are display in Table 5. The Biases are −0.089~0.094 for the fixed effect parameters, −0.063~0.117 for the level-2 variance-covariance component parameters, −0.069~0.105 for the level-3 variance-covariance component parameters. The RMSEs are 0.152~0.311 for the fixed effect parameters, 0.147~0.438 for the level-2 variance-covariance component parameters, 0.132~0.382 for the level-3 variance-covariance component parameters. Furthermore, the Bias and RMSE have a smaller trend with the increase in the number of individuals and items; in other words, increasing the number of individuals and items helps to improve the estimation accuracy of the structural parameters. In summary, the Gibbs sampling algorithm is effective for various numbers of individuals and items, and it can be used to guide practices.

**Table 5:** Evaluating the accuracy of the structure parameters in the simulation 2.

**Number of individuals**	**Number of items**	**Fixed effect** ***γ***		**Level-2 VC** **Σ**_**e**_		**Level-3 VC** *****T*****	
		**Bias**	**RMSE**	**Bias**	**RMSE**	**Bias**	**RMSE**
	40	−0.089	0.031	0.046	0.438	0.064	0.038
1000	100	0.073	0.191	0.078	0.195	−0.037	0.203
	200	0.094	0.174	−0.063	0.160	0.081	0.198
	40	0.056	0.206	0.117	0.319	0.105	0.207
2000	100	0.028	0.167	0.064	0.177	−0.069	0.189
	200	−0.041	0.152	−0.037	0.154	0.021	0.156
	40	0.039	0.231	0.055	0.213	0.032	0.195
3000	100	−0.035	0.189	0.082	0.246	−0.058	0.145
	200	0.017	0.159	0.041	0.147	0.045	0.132

*The VC stands for the abbreviation of variance-covariance*.

## 5. Real Data Analysis−Examining the Correlation Between Different Ability Dimensions and Covariates

To illustrate the applicability of the multidimensional two-parameter normal ogive model in operational large-scale assessments, we consider a data set about students' English achievement test for junior middle schools conducted by NENU Branch, Collaborative Innovation Center of Assessment toward Basic Education Quality at Beijing Normal University. The analysis of the test data will help us to gain a better understanding of the practical situation of students' English academic latent traits and to explore the factors that affect their English academic latent traits. The results of this analysis will be potentially very valuable for development and improvement of educational quality monitoring mechanism in China.

### 5.1. Data Description

The data contain a two-stage cluster sample of 2,029 students in grade 7. These students are from 16 schools, with 121–134 students in each school. In the first stage, the sampling population is classified according to district, and schools are selected at random. In the second stage, students in grade 7 are selected at random from each school. The English test is a test battery consisting of four subscales: vocabulary (40 items), grammar (24 items), comprehensive reading (40 items), and table computing (20 items). All 124 multiple-choice items are scored using a dichotomous format. The Cronbach's alpha coefficients for vocabulary, grammar, reading comprehension and table computing items are 0.942, 0.875, 0.843, and 0.816, respectively. Level-2 and level-3 background covariates of individuals, teacher satisfaction, and school climate (teachers and schools constitute level 3) are measured. At the individual level, gender (0=male, 1=female) and socioeconomic statuses are measured; the latter is measured by the average of two indicators: the father's and mother's education, which are five-point Likert items; scores range from 0 to 8. At the teacher and school levels, teacher satisfaction is measured by 20 five-point Likert items, and school environment from the principal's perspective is measured by 23 five-point Likert items.

#### 5.1.1. Prior Distributions

Based on the setting of priors in the simulation 1, we give the prior distributions of parameters involved in following the real data analysis. The priors of the difficulty parameters and discrimination parameters are set from *b*_*k*_ ~ *N*(0, 1) and $a_{k}={\left(a_{k1},a_{k2},a_{k3},a_{k4}\right)}^{\prime }\sim N\left(0,100{\text{I}}_{4\times 4}\right)\text{I}\left(a_{k}|a_{k1}>0,a_{k2}>0,a_{k3}>0,a_{k4}>0\right),$ *j* = 1, 2, …, 124, where **I**_4×4_ is 4-by-4 identity matrix. The fixed effect ***γ*** follows a uniform distribution *U*(−2, 2). The prior to the variance-covariance matrix of the level-2 ability dimensions is a 4-by-4 identity matrix. The prior to the variance-covariance matrix of the level-3 ***T***_1_, ***T***_2_, ***T***_3_, and ***T***_2_ are set to non-informative priors based on Fox and Glas (2001)'s paper, where *p* (***T***_*q*_) ∝ constant, *q* = 1, 2, 3, 4.

#### 5.1.2. Convergence Diagnosis

The full conditional distribution of Gibbs sampling is run for 20,000 iterations using real data. The trace plots of parameters stabilize after 5,000 iterations. Thus, the first 5,000 iterations are set as the burn-in period. The average over the drawn parameters is calculated after the burn-in period. Moreover, Four chains started at overdispersed starting values are run for monitoring the convergence. The Brook-Gelman ratios are close to 1.2. Therefore, it can be inferred that the estimated parameters are convergent.

### 5.2. Model Selection in Real Data

In the real data example, we consider four dimensions of ability: vocabulary cognitive ability, grammar structure diagnosing ability, reading comprehension ability, and table computing ability. These abilities are affected by individual covariates such as socioeconomic status and gender. The individual can be nested into higher group levels (school), which are affected by group covariates such as teacher satisfactions and school climate from the teachers' perspective. In this current study, we only focus on the specific abilities of four dimensions without the general ability, which is different from Huang and Wang (2014, p. 497, Equation 3)'s ability model with hierarchical structure. According to the above-mentioned DIC model selection method, three models are considered in fitting the real data, in which the DIC can be formulated to choose between models that differ in the fixed and/or random part of the structural model to combine with the measurement model. The multidimensional IRT measurement model is identical to the three candidate models. The structural multilevel model 1 consists of the two level-2 background variables SES and Gender and the level-2 random intercept. The effects of the level-2 background variables SES and Gender are fixed across schools. The structural multilevel part is given by

(5.1) $$Model\text{ 1}\{\begin{array}{l} \theta_{ijq}=\beta_{0jq}+SES_{ij}\beta_{1jq}+Gender_{ij}\beta_{2jq}+e_{ijq},\\ \beta_{0jq}=\gamma_{00q}+u_{0jq},\\ \beta_{1jq}=\gamma_{10q},\\ \beta_{2jq}=\gamma_{20q}. \end{array}$$

Model 2 is extended by including two latent predictors at level 3, Satisfaction and Climate. The effects of the level-2 background variable SES are allowed to vary across schools. The structural multilevel part is given by

(5.2) $$Model\;2\{\begin{array}{l} \theta_{ijq}=\beta_{0jq}+SES_{ij}\beta_{1jq}+Gender_{ij}\beta_{2jq}+e_{ijq},\\ \beta_{0jq}=\gamma_{00q}+Satisfaction_{j}\gamma_{01q}+Climate_{j}\gamma_{02q}+u_{0jq},\\ \beta_{1jq}=\gamma_{10q}+u_{1jq},\\ \beta_{2jq}=\gamma_{20q}. \end{array}$$

Model 3 captures the effects of the level-2 background variables SES and Gender, which are allowed to vary across schools. The structural multilevel part is given by

(5.3) $$Model\text{ 3 }\{\begin{array}{l} \theta_{ijq}=\beta_{0jq}+SES_{ij}\beta_{1jq}+Gender_{ij}\beta_{2jq}+e_{ijq},\\ \beta_{0jq}=\gamma_{00q}+Satisfaction_{j}\gamma_{01q}+Climate_{j}\gamma_{02q}+u_{0jq},\\ \beta_{1jq}=\gamma_{10q}+u_{1jq},\\ \beta_{2jq}=\gamma_{20q}+u_{2jq}. \end{array}$$

Question (1): According to the model selection results, which model is the best to fit the data and how can judge the individual-level regression coefficients be judged as fixed effect or random effect?

The estimated DIC values are presented in Table 6. Model 2 shows that the smallest effective number of model parameters among the three models, which is preferred given the DIC values of the three models. The DIC values of models 2 and 3 are smaller than those of model 1, which can be attributed to the additional latent predictors at level 3, i.e., Satisfaction and Climate. Note that in model 2, the individual random-effect parameters are modeled as group-specific random effects (level-3 Satisfaction and Climate latent predictors), leading to a serious reduction in the effective number of model parameters, which can be inferred from the *P*_*D*_ value in Table 6. The DIC value of model 2 is smaller than that of model 3. The residual *u*_2*jq*_ of the random effect β_2*jq*_ is estimated equal to 0, which is equivalent to fixing the effect of the level-2 background variable Gender across schools.

**Table 6:** Estimated DIC values for the three models fitted to the English test data.

	**P**_**D**_	**$\bar{\text{D}}$**	**DIC**
Model 1	134,470	1,010,030	1,144,500
Model 2	79,065	891,425	970,490
Model 3	81,607	895,073	976,680

### 5.3. Structural Parameter Analysis

Over the past 40 years, a large number of studies have shown that there is a direct relationship between the individuals' language learning ability and the parents' education. For example, Teachman (1987) made use of high school survey data in the United States to explore the influence of family background on childhood education. The results of this study indicated that the parents' occupations, incomes, and educations have a very important impact on children language academic achievement. Moreover, Stern (1983) shows that language is a social mechanism, which needs to be learned in the social environment, even in the biological basis play an important role of mother tongue acquisition, social factors related to children and their parents also play an important role. However, in our study, whether the parents' educational level (SES) has influence on the four kinds of abilities in English learning; the following question will be considered:

Question (2): How will students from different ends of the socioeconomic-status (SES) score in English performance as tested in four types of latent abilities, based on the level-2 gender (GD), level-3 teacher satisfaction (ST) and school climate (CT).

From Tables 7–10, we can find that the estimated fixed effects γ_10*q*_(SES) are 0.642, 0.312, 0.542, and 0.596 for *q* = 1, 2, 3, 4, respectively. It can be observed that students with high SES scores perform better than students with low SES scores, where performance is measured by four types of latent abilities when controlling for the level-2 GD individual covariates and the level-3 ST and CT school covariates. That is, their parents' educational level differs by one unit for the male students from the same class and school. In English learning, vocabulary cognitive ability, the ability to diagnose grammar structure, reading comprehension ability and table computing ability have the differences of 0.642, 0312, 0.542, and 0.596, respectively. The rate of increase in grammatical diagnostic ability (0.312) is markedly smaller than that of the other three kinds of abilities. In addition, compared to male students, the differences in the four dimensions of ability are 0.981, 0.706, 0.874, and 0.330 for female students, respectively. In summary, the education of parents (SES) is responsible for students' English learning abilities. The parents with a high SES values have more prospective awareness in English learning based on their own learning experiences, provide more diversified learning ways, and know how to create a better English learning environment for students. In addition, parents with better education can provide more important learning guidance in English. In general, the better the parents' education, the better they will able to tutor student's English learning.

**Table 7:** Parameter estimation of the multilevel multidimensional IRT model for vocabulary cognitive ability.

	**Vocabulary cognitive ability**		
**Fixed effects**	**EAP**	***SD***	**HPDI**
γ_001_	0.760	0.186	[0.391, 1.137]
γ_011_(*ST*)	0.502	0.143	[0.223, 0.788]
γ_021_(*CT*)	0.225	0.149	[−0.068, 0.520]
γ_101_(*SES*)	0.642	0.128	[0.390, 0.893]
γ_201_(*GD*)	0.339	0.160	[0.025, 0.657]
**Random effects**	**EAP**	**SD**	**HPDI**
$\tau_{001}^{2}$	0.537	0.124	[0.227, 1.200]
$\tau_{011}^{2}$	0.004	0.126	[−0.228, 0.241]
$\tau_{021}^{2}$	−0.006	0.164	[−0.344, 0.383]
$\tau_{111}^{2}\left(SES\right)$	0.247	0.134	[0.112, 0.541]
$\tau_{121}^{2}$	−0.064	0.112	[−0.292, 0.110]
$\tau_{221}^{2}\left(GD\right)$	0.030	0.191	[0.015, 0.043]

*ST, teacher satisfaction; CT, climate; SES, socioeconomic-status; GD, gender. EAP denotes the expected a posteriori estimation. SD denotes the standard deviation. HPDI is the 95% highest posterior density interval*.

**Table 8:** Parameter estimation of the multilevel multidimensional IRT model for diagnosing ability of grammar structure.

	**Vocabulary cognitive ability**		
**Fixed effects**	**EAP**	***SD***	**HPDI**
γ_001_	0.760	0.186	[0.391, 1.137]
γ_011_(*ST*)	0.502	0.143	[0.223, 0.788]
γ_021_(*CT*)	0.225	0.149	[−0.068, 0.520]
γ_101_(*SES*)	0.642	0.128	[0.390, 0.893]
γ_201_(*GD*)	0.339	0.160	[0.025, 0.657]
**Random effects**	**EAP**	**SD**	**HPDI**
$\tau_{001}^{2}$	0.537	0.124	[0.227, 1.200]
$\tau_{011}^{2}$	0.004	0.126	[−0.228, 0.241]
$\tau_{021}^{2}$	−0.006	0.164	[−0.344, 0.383]
$\tau_{111}^{2}\left(SES\right)$	0.247	0.134	[0.112, 0.541]
$\tau_{121}^{2}$	−0.064	0.112	[−0.292, 0.110]
$\tau_{221}^{2}\left(GD\right)$	0.030	0.191	[0.015, 0.043]

*ST, teacher satisfaction; CT, climate; SES, socioeconomic-status; GD, gender. EAP denotes the expected a posteriori estimation. SD denotes the standard deviation. HPDI is the 95% highest posterior density interval*.

**Table 9:** Parameter estimation of the multilevel multidimensional IRT model for reading comprehension ability.

	**Reading comprehension ability**		
**Fixed effects**	**EAP**	***SD***	**HPDI**
γ_003_	0.919	0.187	[0.548, 1.293]
γ_013_(*ST*)	0.332	0.148	[0.041, 0.624]
γ_023_(*CT*)	0.081	0.168	[−0.249, 0.417]
γ_103_(*SES*)	0.542	0.118	[0.308, 0.780]
γ_203_(*GD*)	0.232	0.155	[−0.070, 0.544]
**Random effects**	**EAP**	***SD***	**HPDI**
$\tau_{003}^{2}$	0.535	0.111	[0.223, 1.220]
$\tau_{013}^{2}$	0.040	0.198	[−0.156, 0.275]
$\tau_{023}^{2}$	−0.024	0.153	[−0.342, 0.264]
$\tau_{113}^{2}\left(SES\right)$	0.207	0.133	[0.091, 0.456]
$\tau_{123}^{2}$	0.004	0.089	[−0.170, 0.182]
$\tau_{223}^{2}\left(GD\right)$	0.037	0.177	[0.027, 0.052]

*ST, teacher satisfaction; CT, climate; SES, socioeconomic-status; GD, gender. EAP denotes the expected a posteriori estimation. SD denotes the standard deviation. HPDI is the 95% highest posterior density interval*.

**Table 10:** Parameter estimation of the multilevel multidimensional IRT model for table computing ability.

	**Table computing ability**		
**Fixed effects**	**EAP**	***SD***	**HPDI**
γ_004_	0.255	0.130	[−0.003, 0.514]
γ_014_(*ST*)	0.039	0.104	[−0.165, 0.246]
γ_024_(*CT*)	0.295	0.101	[0.099, 0.498]
γ_104_(*SES*)	0.596	0.126	[0.351, 0.849]
γ_204_(*GD*)	−0.266	0.120	[−0.506, -0.026]
**Random effects**	**EAP**	***SD***	**HPDI**
$\tau_{004}^{2}$	0.447	0.144	[0.201, 0.970]
$\tau_{014}^{2}$	0.082	0.084	[−0.043, 0.269]
$\tau_{024}^{2}$	−0.041	0.100	[−0.223, 0.098]
$\tau_{114}^{2}\left(SES\right)$	0.226	0.106	[0.101, 0.485]
$\tau_{124}^{2}$	−0.014	0.069	[−0.160, 0.114]
$\tau_{224}^{2}\left(GD\right)$	0.022	0.102	[0.015, 0.035]

*ST, teacher satisfaction; CT, climate; SES, socioeconomic-status; GD, gender. EAP denotes the expected a posteriori estimation. SD denotes the standard deviation. HPDI is the 95% highest posterior density interval*.

Etaugh and Bridges (2003), Li (2005), and Burstall (1975) found that females were better than males in most of the language tasks (vocabulary, reading, grammar, spelling and writing), and the difference in language ability appeared earlier than other cognitive abilities. In infancy, females show more linguistic advantages than males, and they speak more fluently, and have a richer vocabulary. To about 11 years old, they are not only good at simple spelling, but also are able to do more complicated writing tasks. In schools, teachers have found that females do better in reading comprehension, and they are less likely to have reading problems, including reading barriers. However, whether or not have the above conclusions in this study, next the following issues will be considered:

Question (3): What relationship exists between males and females' performances in different latent abilities by controlling for SES, ST and CT?

Results from Tables 7–10 show that for male and female students from the same class and school with the same SES scores, female students' performances of vocabulary cognitive ability, the ability to diagnose grammar structure and reading comprehension ability are higher than those of male students 0.339, 0.394, 0.232. However, male students have a 0.266 advantage over female students in table computing ability. This empirical study yields almost identical conclusions for Etaugh and Bridges (2003). That is, male and female students, who have the same SES scores in the same class and school, have a great difference in the acquisition of English proficiency. Moreover, in terms of vocabulary cognition, grammatical structure analysis, reading comprehension it can be seen that females are better than males at vivid memory and mechanical memory is stronger than males. However, compared to females, males are markedly better than females at logical reasoning, deductive induction, and computing ability. In addition, according to gender difference in English learning of middle school students, the improving measure of learning from others' strong points to offset one' own weakness mainly covers: first, either teachers of students should properly understand the gender difference; second, to strengthen female students' training of logical thinking; third, to widen female students' reasoning computing ability; fourth, for the male students, to develop their vivid memory through a variety of teaching methods. These four points should be parallel in structure.

Question (4): What effects, if any, are seen with different teachers' or schools' effects (covariates)?

For male students who have the same SES scores from different schools, if the difference in teacher satisfaction is a unit, the difference in vocabulary cognitive ability, the ability to diagnose grammar structure and reading comprehension ability are 0.502, 0.335, and 0.331, respectively. However, the difference in the table computing ability is very small for 0.039. Teachers' factor has an important effect on students' cognitive ability, the ability to diagnose grammar structure and reading ability. On the contrary, the table computing ability has little impact.

This study indicates that the middle school teachers with high teacher satisfactions have a strong sense of responsibility, can be filled with enthusiasm in the work of education and teaching, and inspire students' learning motivation. This results in a great improvement in the students' vocabulary cognitive ability, the ability to analyze grammatical structure and reading comprehension ability owing to teachers' teaching attitude and responsibility. However, the margin of the improvement for the table computing ability is small. It is possible to play a decisive role in the students' internal factors as compared with the teachers' external factors.

As we know, people are the product of the environment. The environment has a great impact on cognition, emotion and behavior intention. Different people live in different environments so that there is a huge difference in cognition, emotion and behavior intention. Similarly, in English teaching, are whether or not the performances identical for different schools' effects (school climate)? If not, what are the effects?

The estimated results for school climate effects γ_02*q*_ are 0.225, 0.081, 0.086, and 0.295 for *q* = 1, 2, 3, 4, respectively. The performances associated with vocabulary cognitive ability and table computing ability are markedly affected by the level-3 CT covariates, whereas the ability to diagnose grammar structure and reading comprehension ability are not markedly affected when controlling for the level-2 SES and GD individual covariates and the level-3 ST school covariates. Analysis of the level-3 variance components reveals that the values of $\tau_{11q}^{2}$(SES) are markedly different from 0, and their estimates are 0.247, 0.272, 0.207, and 0.226 for *q* = 1, 2, 3, 4, respectively. This result illustrates that the effect of SES varies from school to school. In addition, the $\tau_{22q}^{2}$(GD) values are not markedly different from 0. In addition, according to the DIC model selection results, model 2 shows the best fit to the real data when β_2*jq*_ are defined as fixed effects. The estimation results show that the proportion of females to males does not vary among schools. The estimation covariance between the random effects $\tau_{01q}^{2}$, $\tau_{02q}^{2}$, and $\tau_{12q}^{2}$ are all not markedly different from 0. It can be concluded that the random effects are independent of each other for each type of ability. All estimated parameters are shown in Tables 7–10.

### 5.4. Item Test Dimension Evaluation

Question (5): Is it possible to use a measurement tool to determine whether items' factor patterns correlate to the subscales of the test battery? In particular, will the four subtests of the test battery be discernable according to the discrimination parameters on the four dimensions?

**Figure 1:**
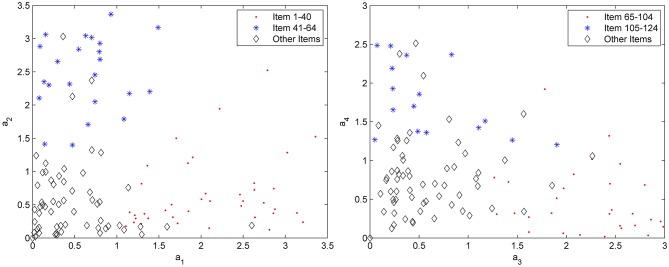
Parameters of estimation *a*_*k*1_, *a*_*k*2_, *a*_*k*3_, and *a*_*k*4_ for subscale 1 (items 1–40), subscale 2 (items 41–64), subscale 3 (items 65–104), and subscale 4 (items 105–124).

A test battery contains four subtests, which consist of items of measuring four dimensional abilities, and a type of latent ability can be measured mainly by a subtest. It can be observed that the EAP estimates of the discrimination parameters are plotted to determine whether the items' factor patterns reflect the subtest of the test battery in Figure 1. In the left-hand panel of Figure 1, the discrimination parameters of the first two dimensions are plotted for subtest 1 (items marked by a dot) and subtest 2 (items marked by a star), and the other items are marked by a diamond. It can be observed that the items of subtest 1 (1–40 item) have a high factor loading on the first dimension and a low factor loading on the second dimension, and the items of subtest 2 (41–64 item) have a high factor loading on the second dimension and a low factor loading on the first dimension. The other items do not vary appreciably between the two dimensions. The right-hand panel of Figure 1 shows the pattern of the discrimination parameters of the third and fourth subtests on the third and fourth dimensions. The items of subtest 3 (65–104 item) have a high factor loading on the third dimension and a low factor loading on the fourth dimension, and the items of subtest 4 (105–124 item) have a high factor loading on the fourth dimension and a low factor loading on the third dimension. The overall pattern of the discrimination parameters fit the test battery quite well, demonstrating that each dimension is identified by items of one subtest.

## 6. Concluding Remarks

In this study, we mainly focus on constructing a multilevel multidimensional model to fit the hierarchical dataset about a large-scale English achievement test. Particular attention is given to assessing the correlation between multiple latent abilities and covariates.

In view of the characteristics of the test structure (i.e., (1) the students are nested within classes or schools; (2) the binary response consists of several subtests and each subtest measures a distinct latent trait), we extend the measurement model developed by Fox and Glas (2001) and Kamata (2001) to the multidimensional case by replacing their unidimensional IRT model with a multidimensional normal ogive model. The numerical results show that the multidimensional IRT model is appropriate for modeling the measurement model. It can accurately model the item/person interaction and utilize the correlations between subtests to increase the measurement precision of each subtest.

From what has been using the above empirical data, we may safely draw valuable conclusions to provide guidance for the future English teaching. Socioeconomic status (SES) has a positive impact on the abilities of four dimensions. That is, the higher families' SESs, the better performances in the four dimensional abilities. In addition, the study also found that students of different genders do not demonstrate the same level of expertise in English skills are expert in the English skills are not the same. Female students are good at the items related to the memory of the image and mechanical memory, such as the vocabulary, grammar and reading comprehension; but the male students have the advantage in reasoning calculation. Therefore, teachers should adjust the teaching methods based on the gender differences so that he or she can acquire the ability to overcome their own deficiency. Teachers' satisfaction as level 3 teacher covariate markedly impacts English table computing ability. It is possible to play a decisive role in the students' internal factors as compared with the teachers' external factors. Finally, the impact of the school climate factor on students' grammatical structure analysis and reading comprehension is not very obvious, and the specific reasons are to be studied later.

In the future studies, the correlations between schools at the level-3 should be taken into consideration. For example, the different secondary schools which are located in the same district may share a common education resources. In addition, the measurement model can be improved by considering polytomous item response theory model to analyze ordinal response data with more information. As an extension of this paper, the polytomous response model associated with the multilevel models can be used to help evaluate the multiple latent abilities, which may be more suitable for the current complex situation of educational and psychological research. In the field of estimation method, Bayesian estimation method will face serious challenges when the number of examinees or the number of items, or MCMC sample size are substantially increased. Therefore, the proposal of efficient Bayesian algorithm and the development of easy-to-use software package are also important research focus in the later period.

## Data Availability Statement

The datasets for this manuscript are not publicly available because Data from NENU Branch, Collaborative Innovation Center of Assessment toward Basic Education Quality at Beijing Normal University has signed a confidentiality agreement. Requests to access the datasets should be directed to taoj@nenu.edu.cn.

## Author Contributions

FC completed the writing of the article. JL and JT provided key technical support. JZ provided original thoughts and article revisions.

### Conflict of Interest

The authors declare that the research was conducted in the absence of any commercial or financial relationships that could be construed as a potential conflict of interest.

## Supplementary Material

Figure S1 Trace plot of a_9,1_.

Figure S2 Trace plot of a_9,2_.

Figure S3 Trace plot of b_9_.

Figure S4 Trace plot of a_26,1_.

Figure S5 Trace plot of a_26,2_.

Figure S6 Trace plot of b_26_.

Figure S7 Trace plots of the fixed effects in the first dimension.

Figure S8 Trace plots of the fixed effects in the second dimension.

Figure S9 Trace plot of $\sigma_{e1}^{2}$.

Figure S10 Trace plot of σ_*e*1*e*2_.

Figure S11 Trace plot of $\sigma_{e2}^{2}$.

Figure S12 Trace plot of τ_001_.

Figure S13 Trace plot of τ_002_.

Figure S14 Trace plot of τ_011_τ_101_.

Figure S15 Trace plot of τ_012_τ_102_.
