# Supplementary figures and images for: Exploring the Correlation Between Multiple Latent Variables and Covariates in Hierarchical Data Based on the Multilevel Multidimensional IRT Model
Source: Front Psychol. 2019 Oct 25;10:2387. doi: 10.3389/fpsyg.2019.02387 (PMC6823212; doi:10.3389/fpsyg.2019.02387)

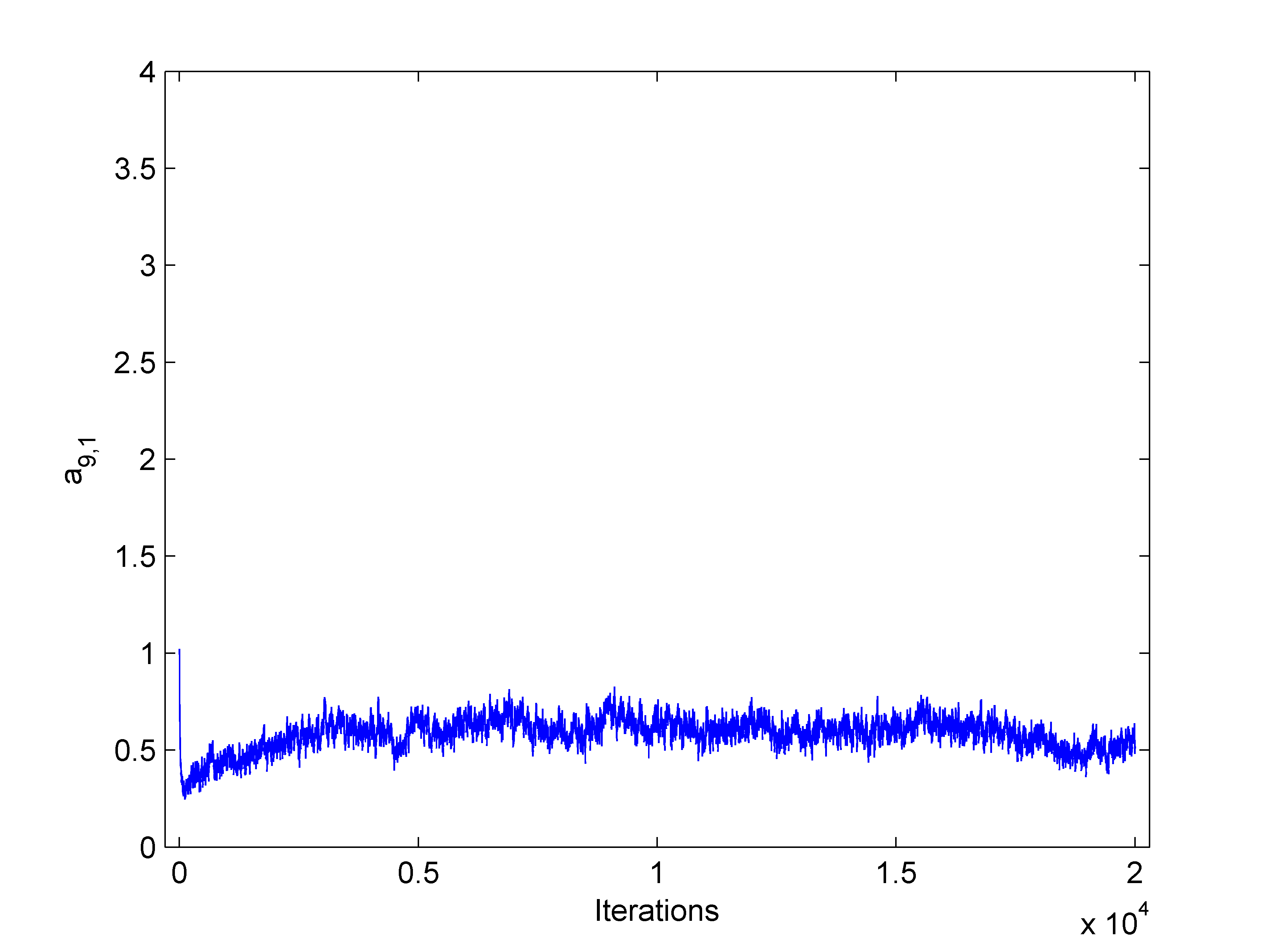

Supplement: Figure S1 — Trace plot of a9,1. [file Image_1.PNG]

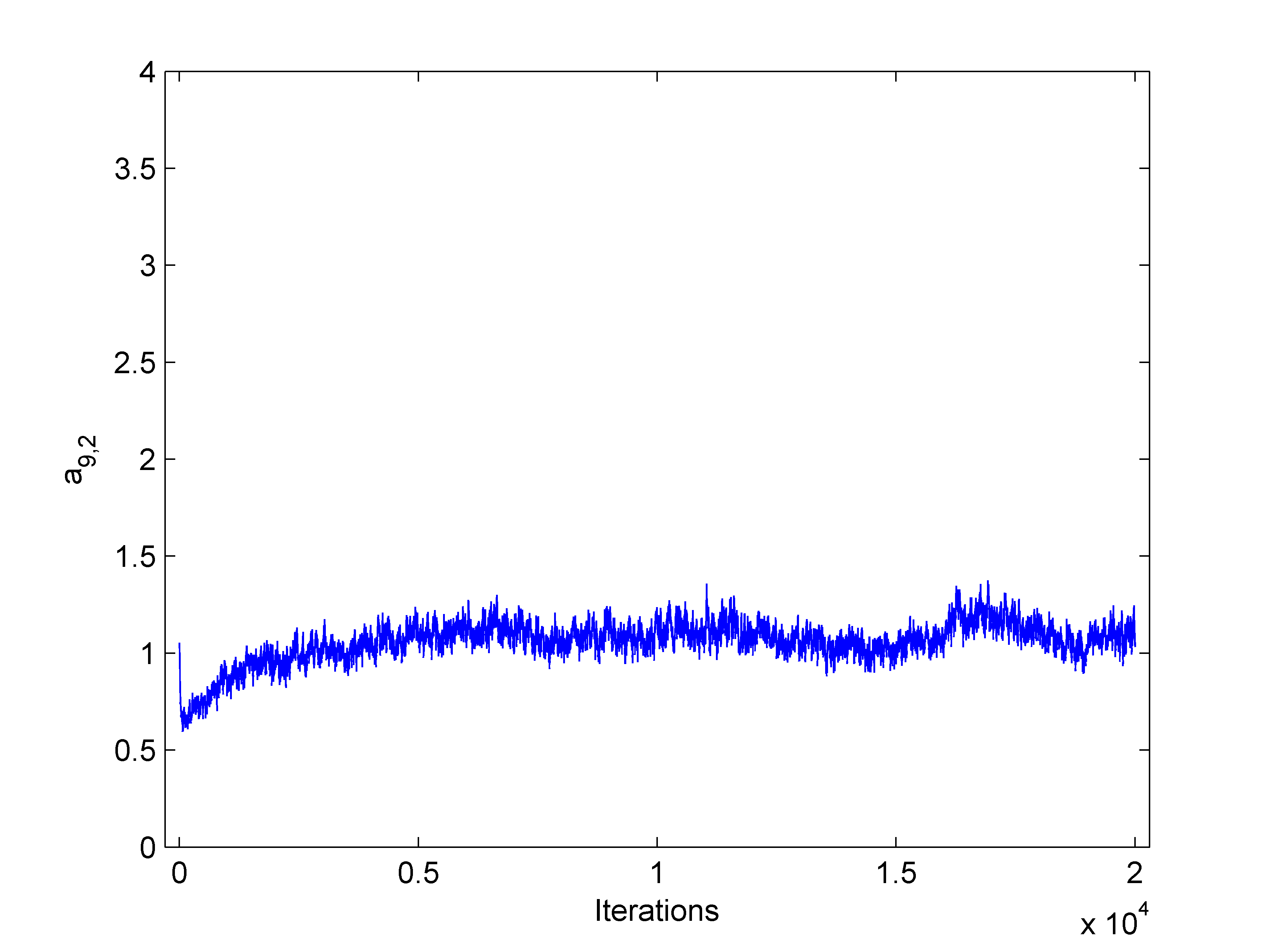

Supplement: Figure S2 — Trace plot of a9,2. [file Image_2.PNG]

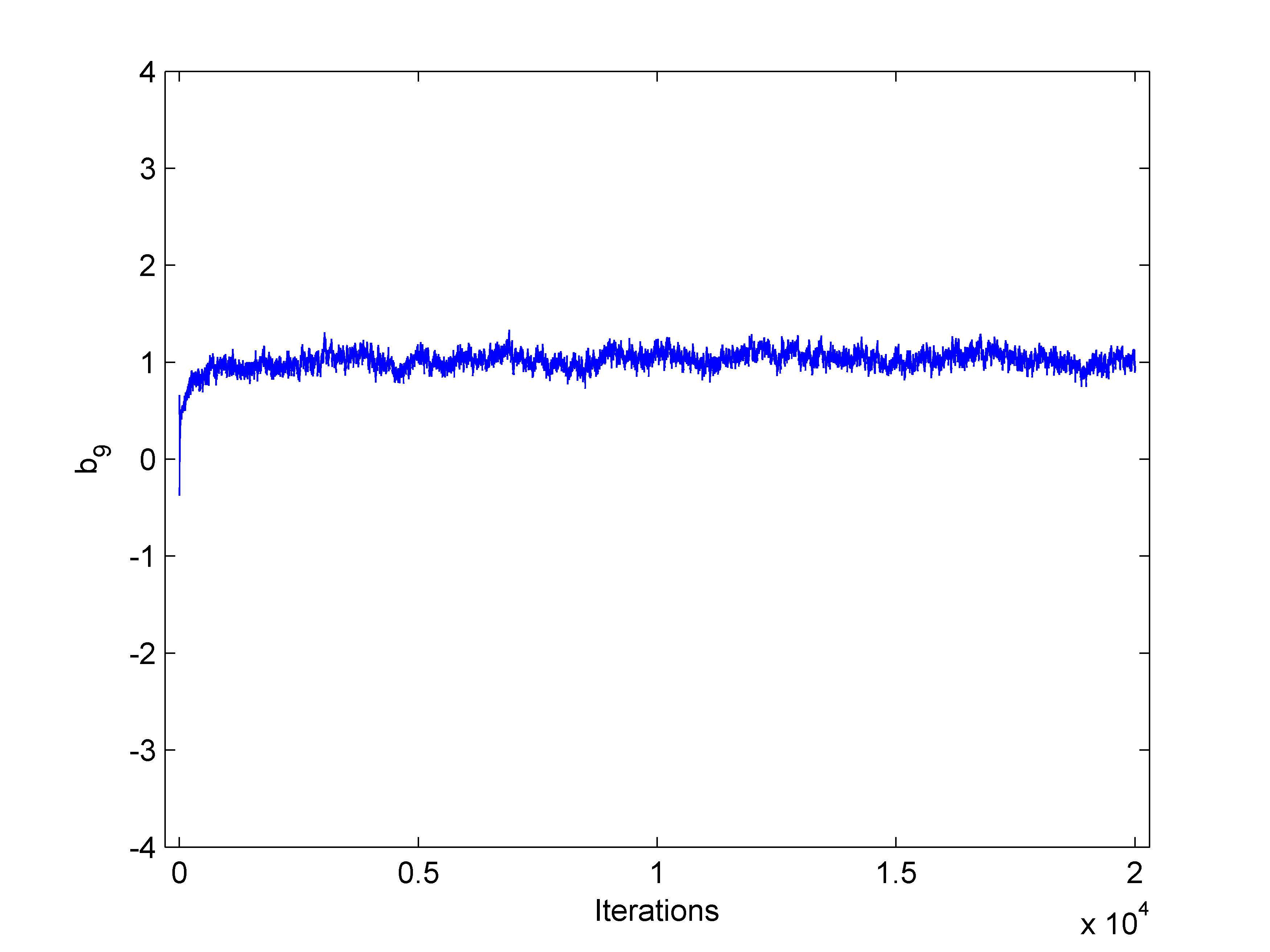

Supplement: Figure S3 — Trace plot of b9. [file Image_3.PNG]

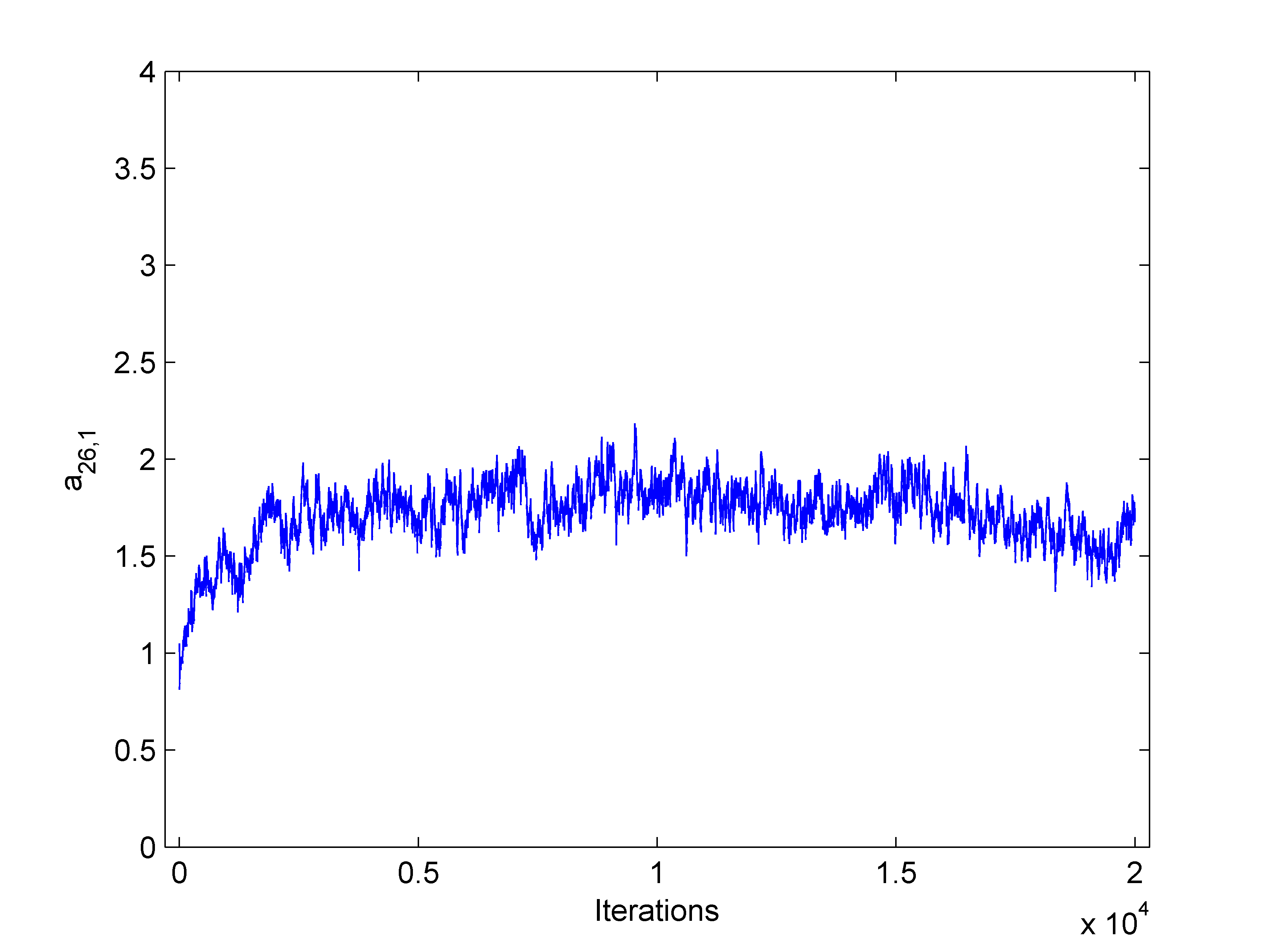

Supplement: Figure S4 — Trace plot of a26,1. [file Image_4.PNG]

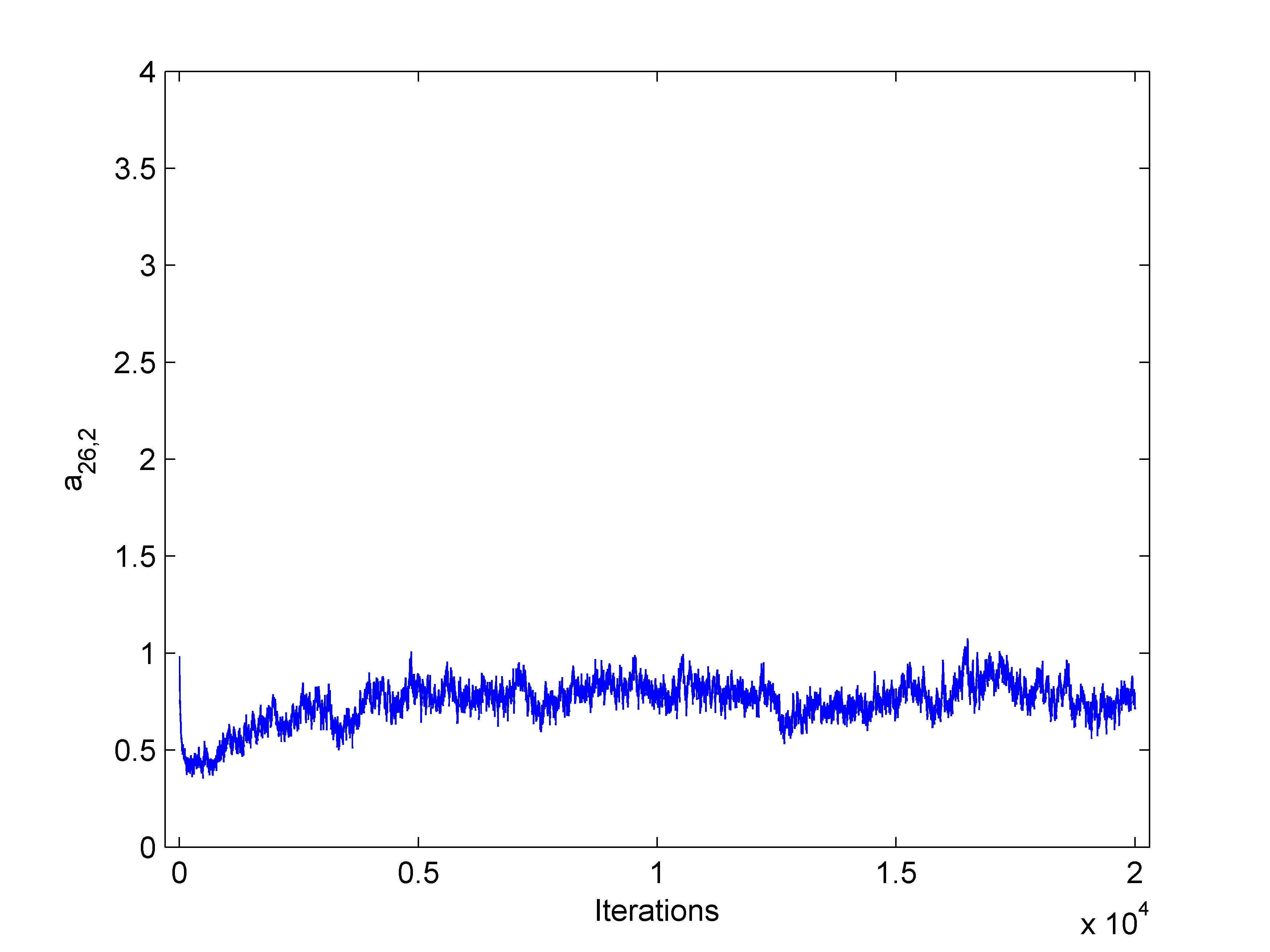

Supplement: Figure S5 — Trace plot of a26,2. [file Image_5.PNG]

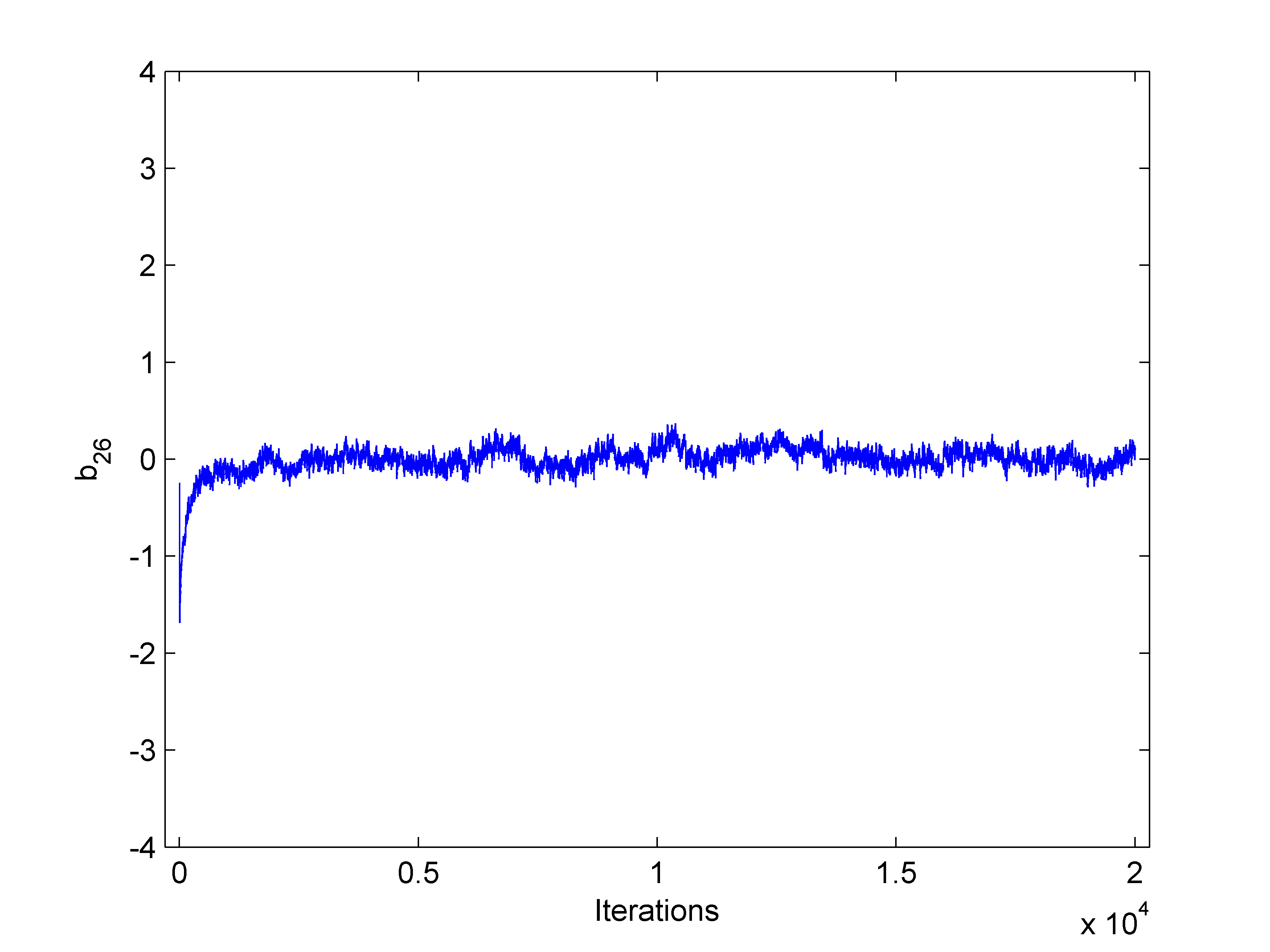

Supplement: Figure S6 — Trace plot of b26. [file Image_6.PNG]

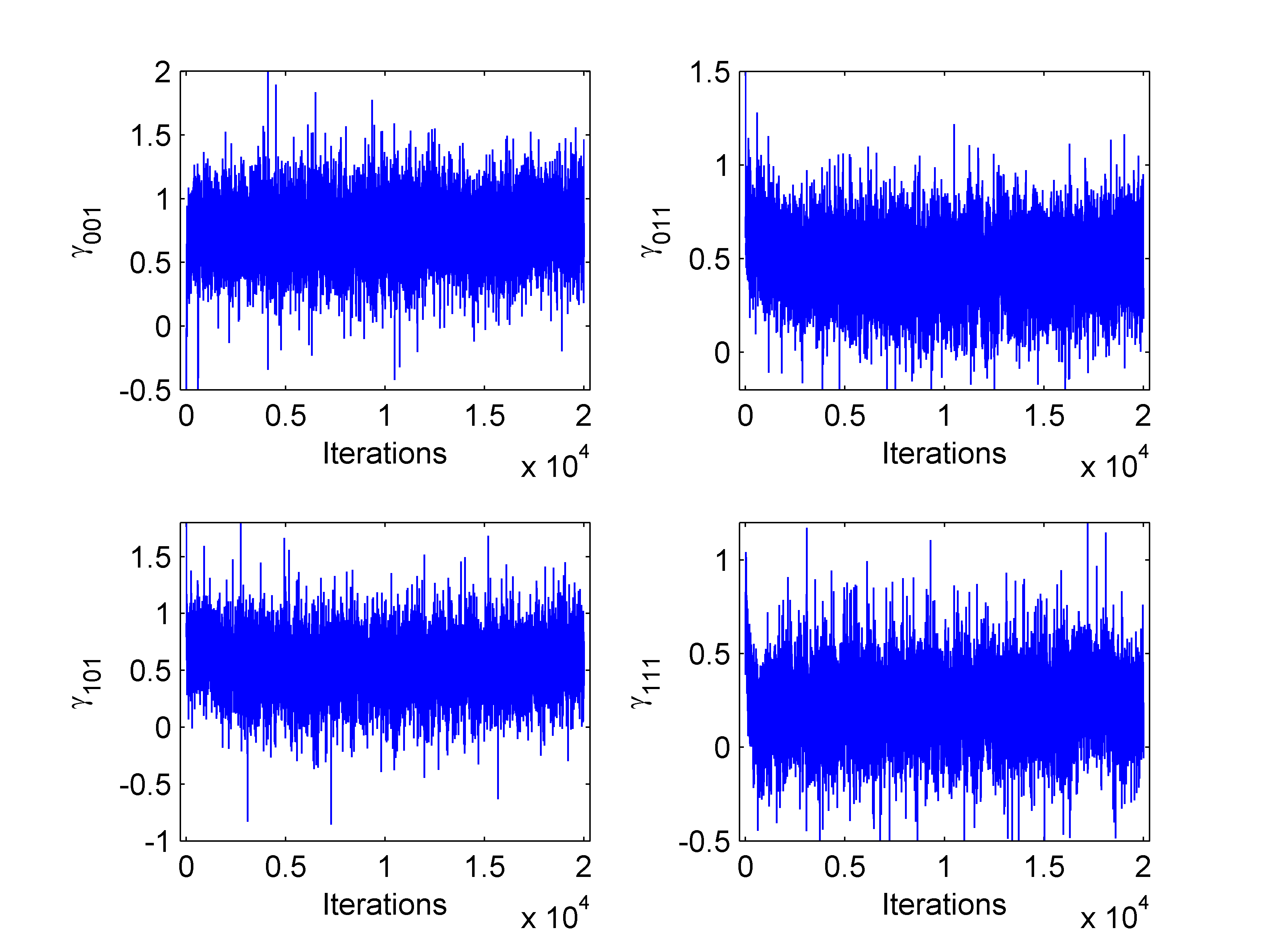

Supplement: Figure S7 — Trace plots of the fixed effects in the first dimension. [file Image_7.PNG]

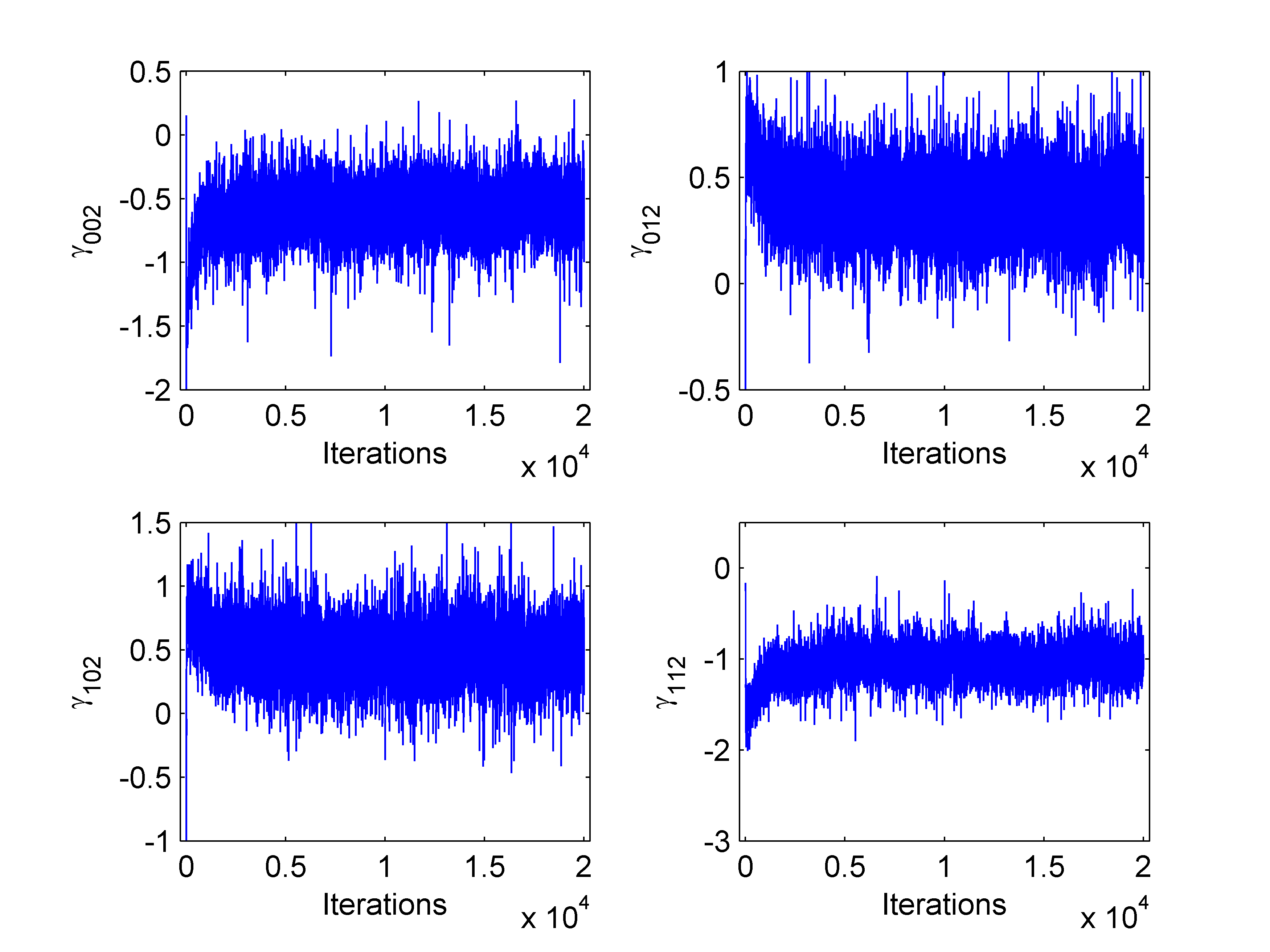

Supplement: Figure S8 — Trace plots of the fixed effects in the second dimension. [file Image_8.PNG]

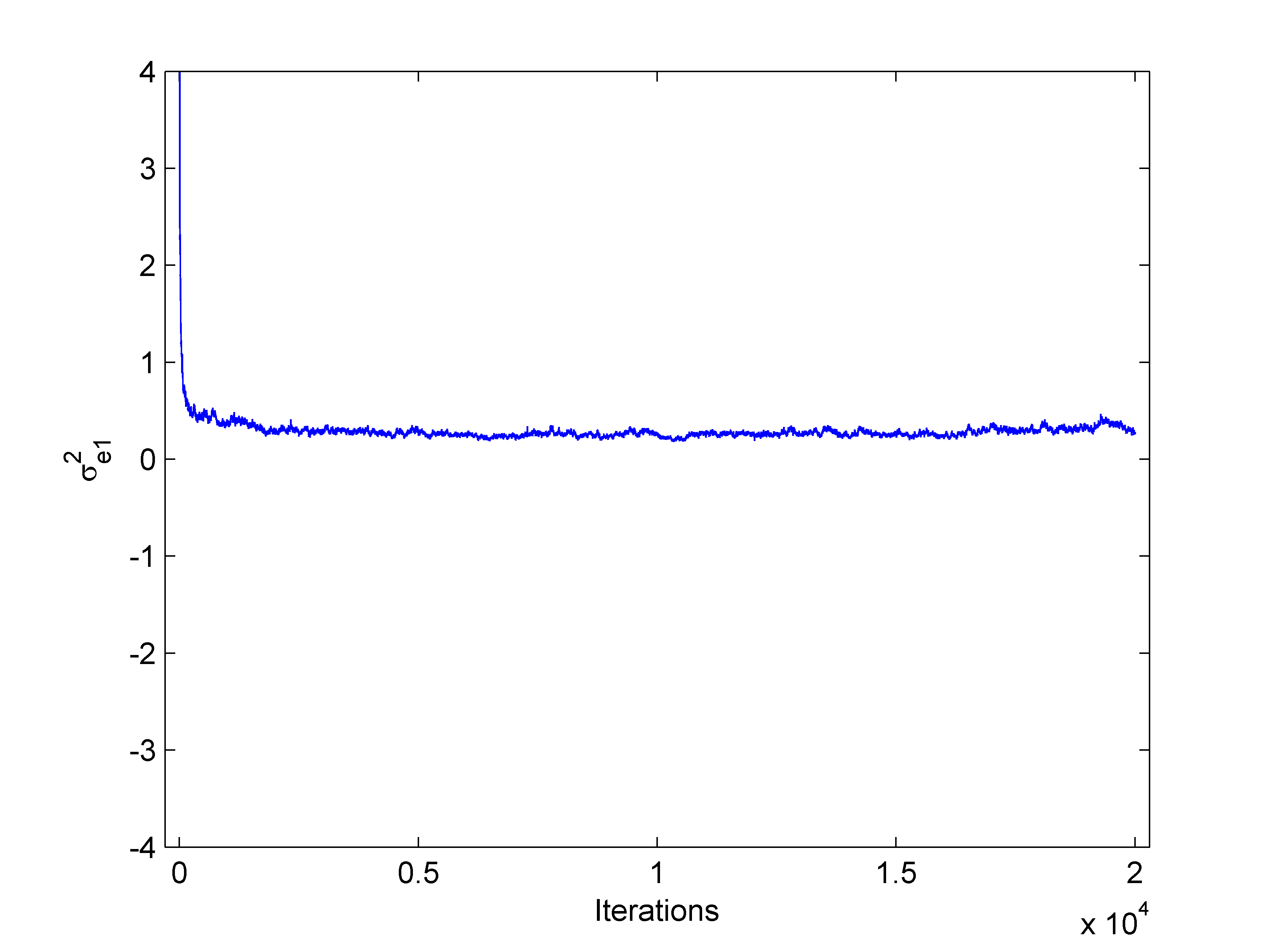

Supplement: Figure S9 — Trace plot of σe12. [file Image_9.PNG]

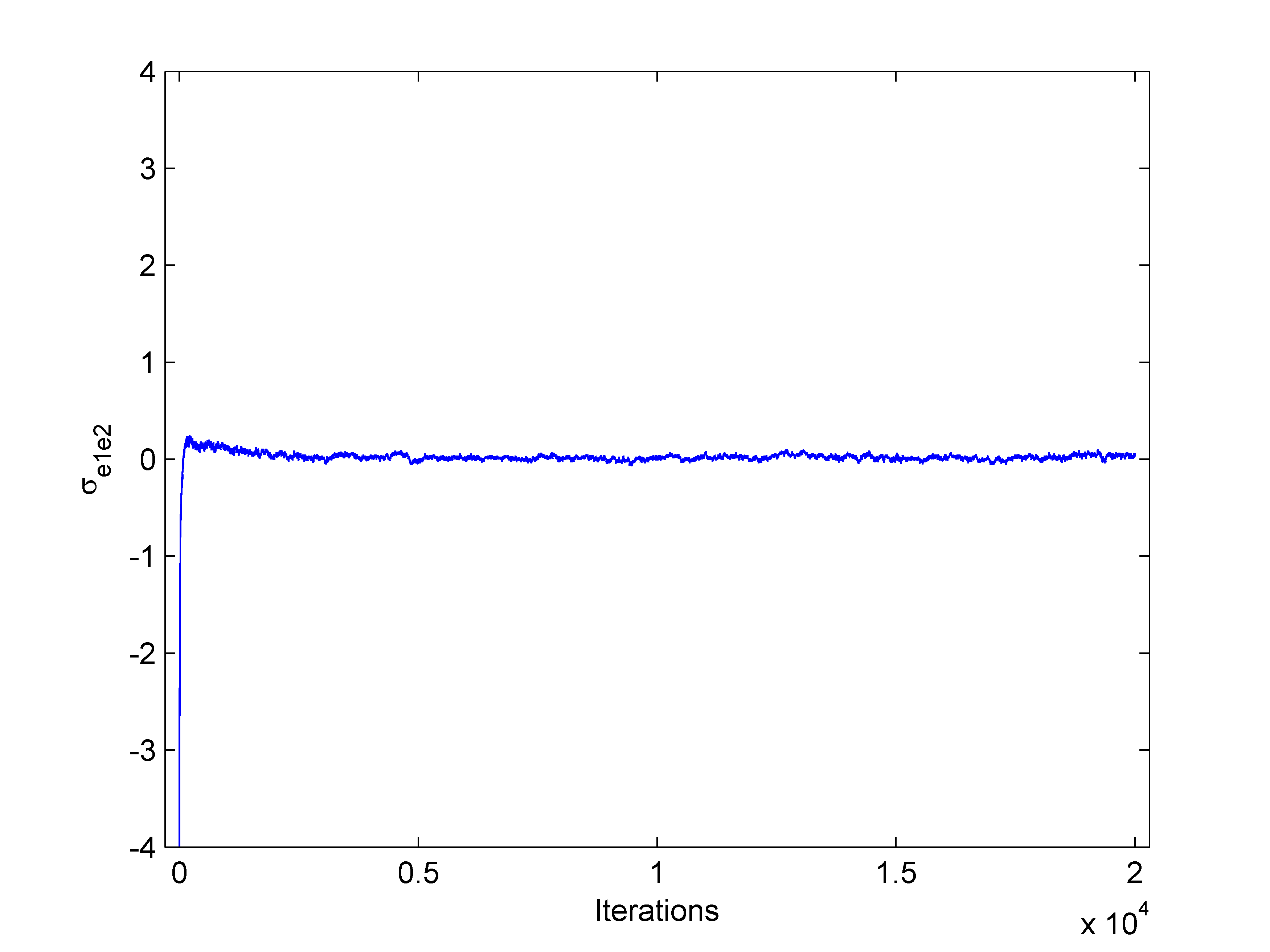

Supplement: Figure S10 — Trace plot of σe1e2. [file Image_10.PNG]

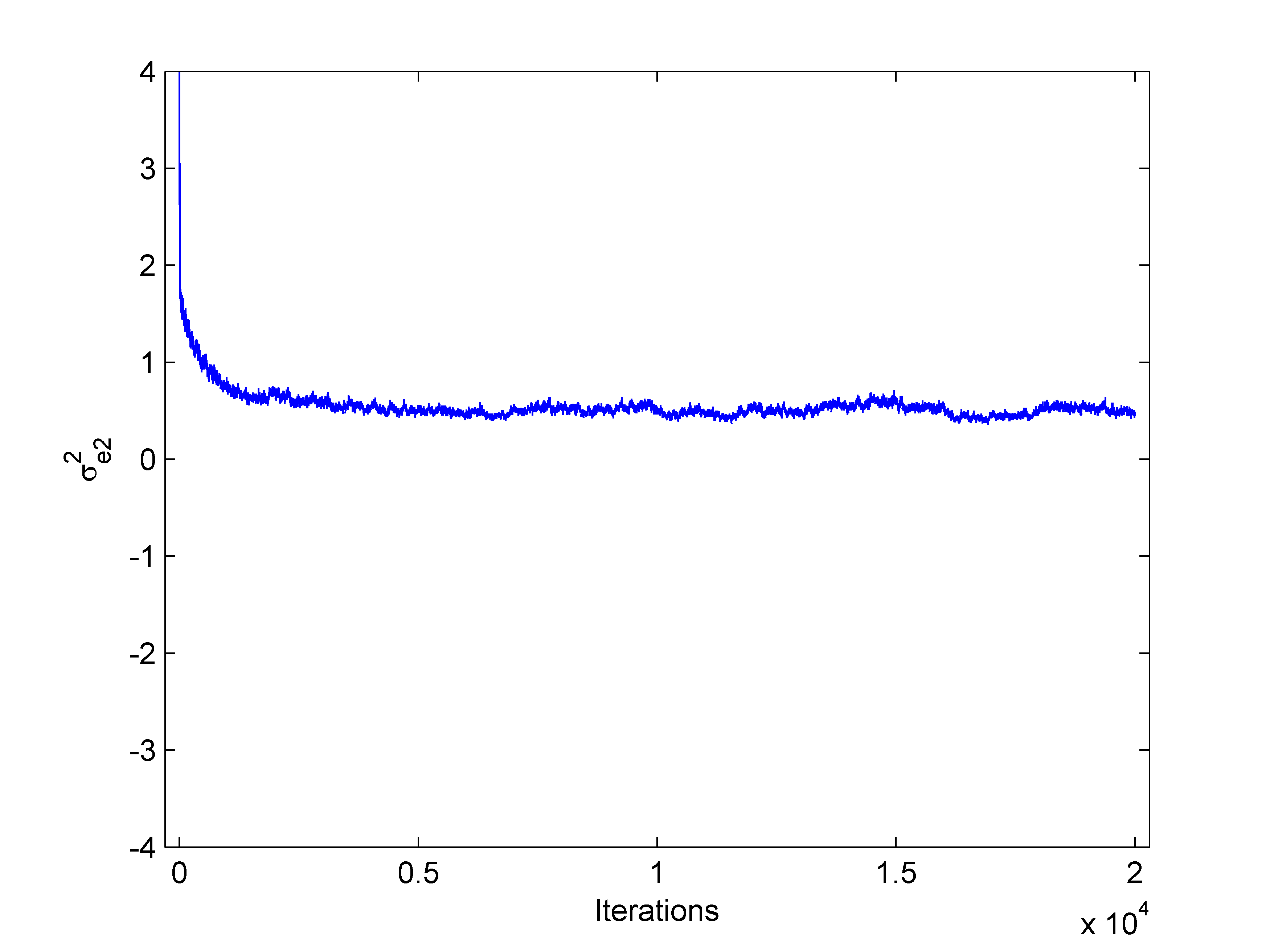

Supplement: Figure S11 — Trace plot of σe22. [file Image_11.PNG]

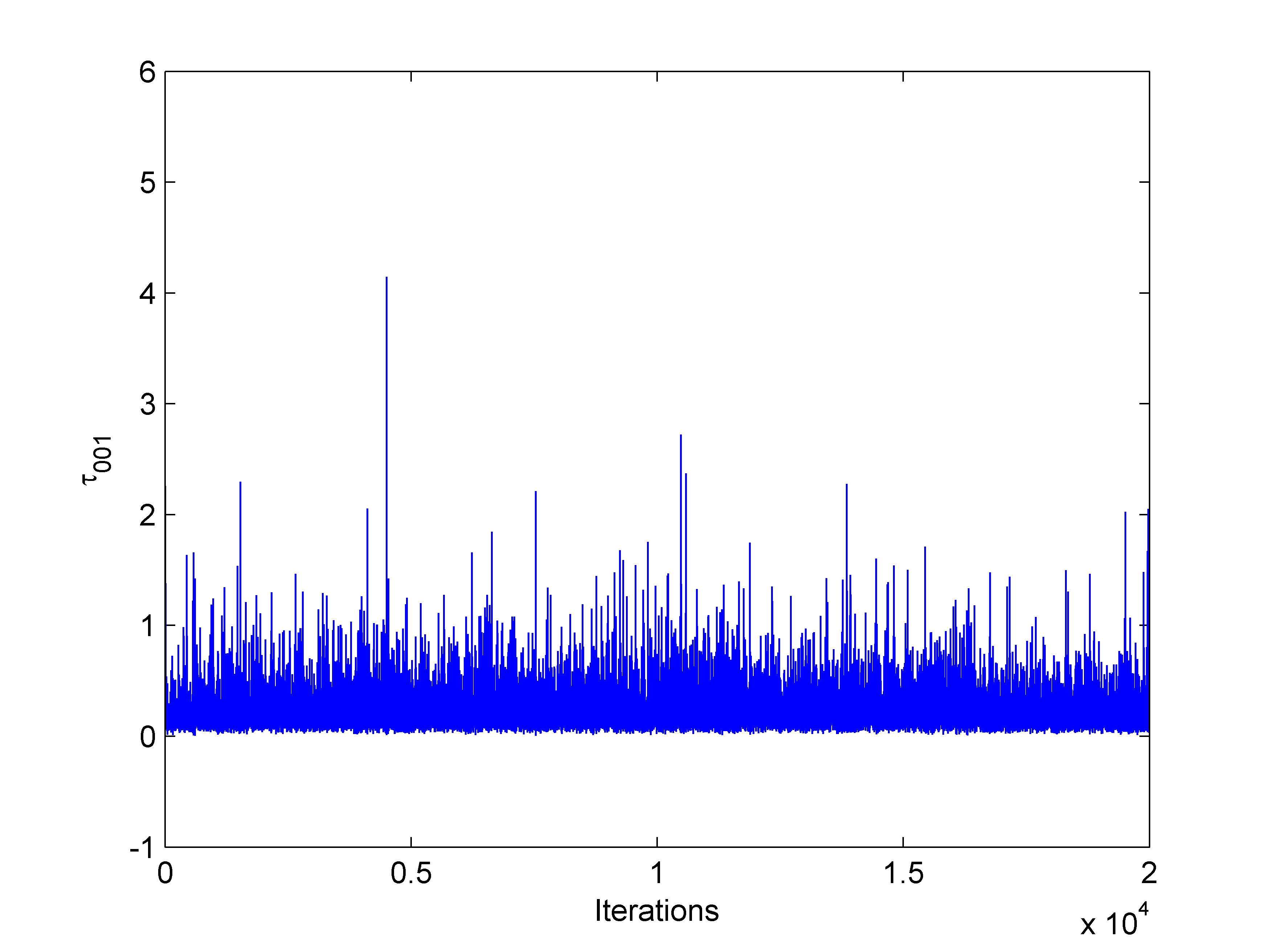

Supplement: Figure S12 — Trace plot of τ001. [file Image_12.PNG]

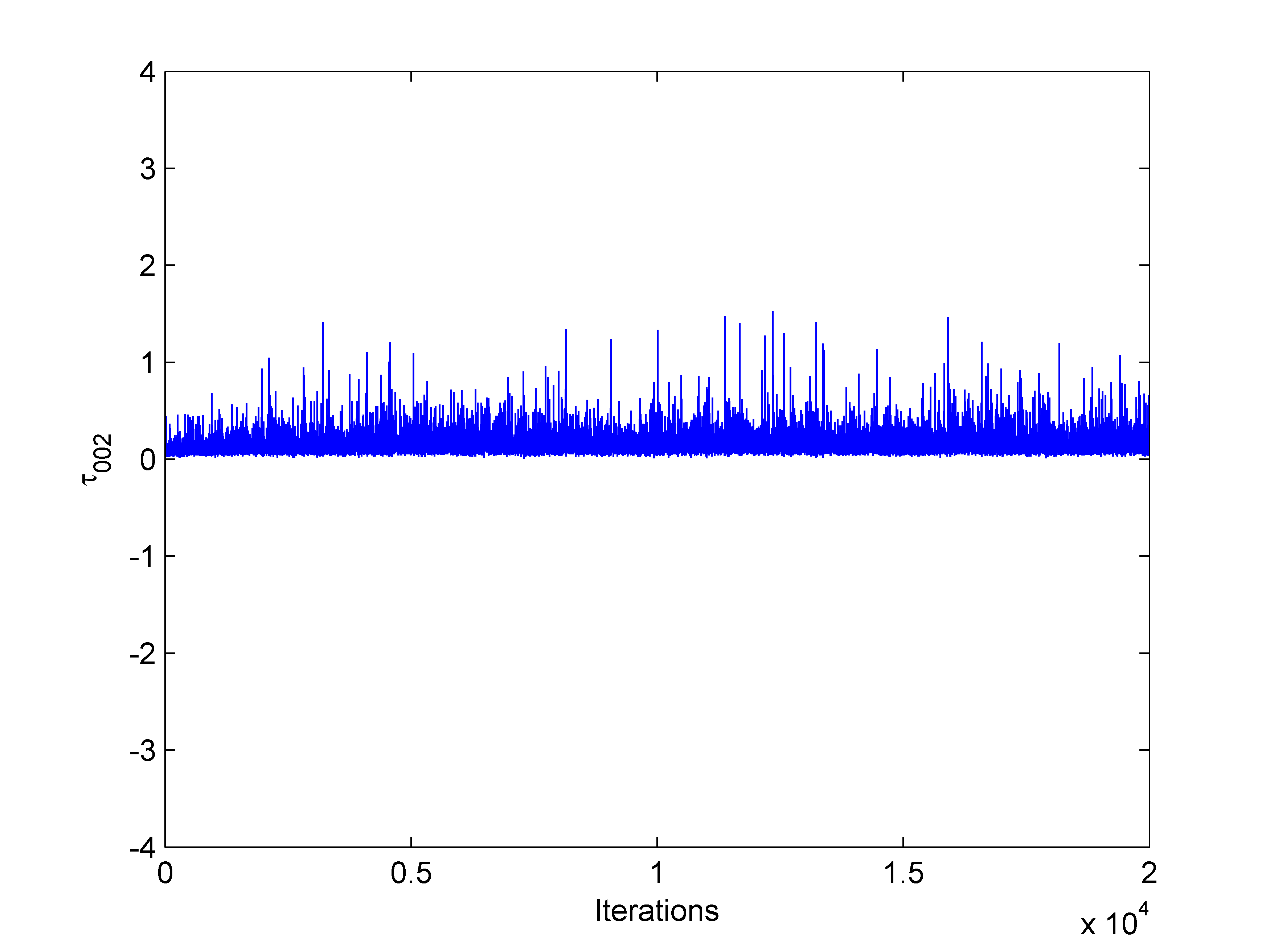

Supplement: Figure S13 — Trace plot of τ002. [file Image_13.PNG]

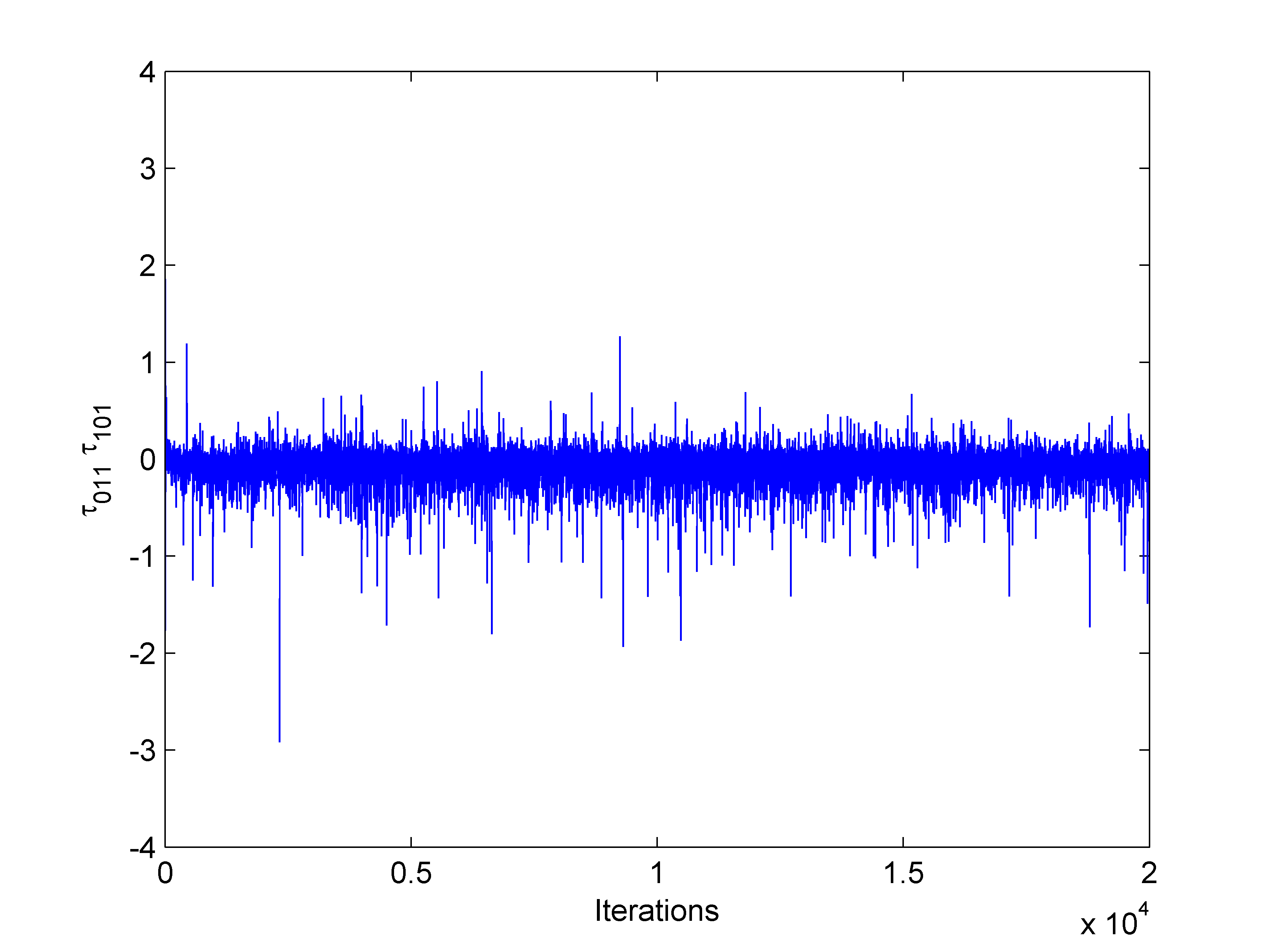

Supplement: Figure S14 — Trace plot of τ011τ101. [file Image_14.PNG]

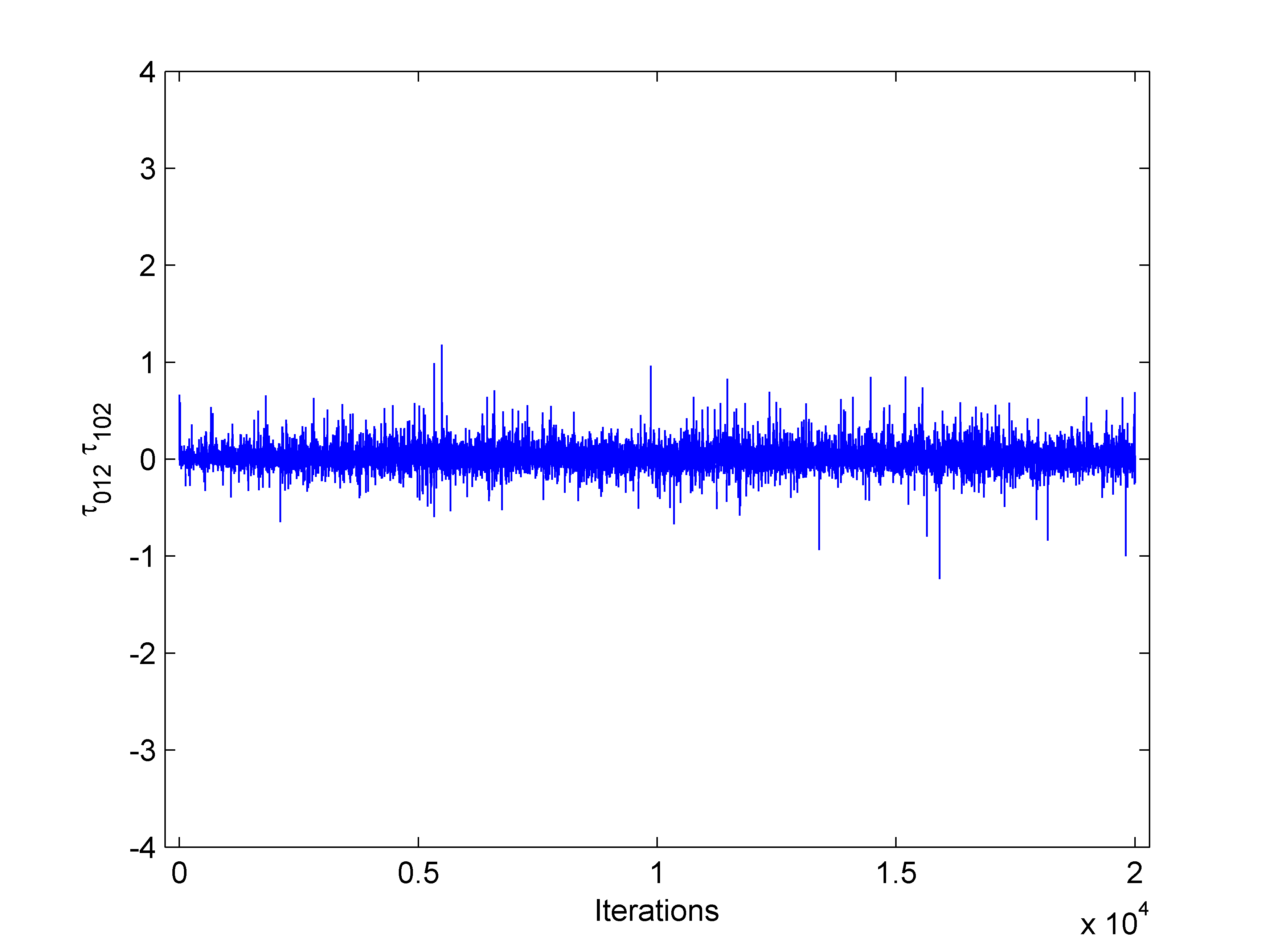

Supplement: Figure S15 — Trace plot of τ012τ102. [file Image_15.PNG]
